# Knowledge, Attitude, and Self-Reported Practice Towards Measures for Prevention of the Spread of COVID-19 Among Australians: A Nationwide Online Longitudinal Representative Survey

**DOI:** 10.3389/fpubh.2021.630189

**Published:** 2021-06-02

**Authors:** Joanne Enticott, William Slifirski, Kim L. Lavoie, Simon L. Bacon, Helena J. Teede, Jacqueline A. Boyle

**Affiliations:** ^1^Monash Centre for Health Research and Implementation, School of Public Health and Preventive Medicine, Monash University, Clayton, VIC, Australia; ^2^Monash Partners Academic Health Science Centre, Clayton, VIC, Australia; ^3^Department of Psychology, University of Quebec at Montreal, Montreal, QC, Canada; ^4^Montreal Behavioral Medicine Centre, Centre Integrée Universitaire de Santé et Services Sociaux du Nord de l'Ile de Montréal (CIUSSS-NIM), Montreal, QC, Canada; ^5^Department of Health, Kinesiology and Applied Physiology, Concordia University, Montreal, QC, Canada; ^6^School of Public Health and Preventive Medicine, Monash University, Melbourne, VIC, Australia

**Keywords:** COVID-19, representative survey Australia, representative survey, public health behaviour, health policies

## Abstract

**Objective:** To assess and share learnings on the motivators and behavioural adherence across sex and age to evolving strategies in public policy to prevent the spread of SARS-CoV-2 at the end of a first COVID-19 wave and the beginning of a second COVID-19 wave in Australia.

**Design and Setting:** A national longitudinal survey using a framework based on evidence-based behaviour change models. The survey was administered to a national sample representative across sex, age and location was undertaken at two time points: May 1st to 5th, 2020, and July 1st to 7th, 2020.

**Results:** Overall 2,056 surveys were completed across the first and second rounds, with 63% (1,296/2,056) completing both. Age range was 18–99 years (median 53, IQR: 34–64). Suboptimal physical distancing and self-quarantining if unwell/diagnosed was reported in one in four respondents and not getting a test at onset of symptoms reported in one in three. Those non-adherent to all three behaviours (19%, 60/323), were mainly male, younger, lived in major cities and reported fewer concerns or motivators to change behaviour. Overall, government lockdown measures were considered very important by 81% (835/1,032) and appropriate by 75% (772/1,029).

**Conclusions:** Prior to the suppression of a second COVID-19 wave, a significant minority of Australians reported suboptimal behavioural adherence to vital policy strategies to limit SARS-CoV-2 spread, mostly young adults and men. Successful wave 2 suppression required consistent communication from political and health leaders and supportive public health and economic strategies. Additional lockdown and punitive strategies were needed in Victoria and were generally well-supported and adhered to. To limit subsequent lockdown, this work reinforces the need for a mix of communication around saving lives of the vulnerable, and other strategies targeting high risk groups, facilitation of easy testing and minimisation of financial impacts.

## What Is Already Known?

- Governments globally have been forced to implement extraordinary public health measures to control the spread of disease and prevent significant loss of life, and these interventions require substantial and sustained behaviour change, and come at significant personal, psychosocial and economic costs.
- If we are to be successful in containing SARS-CoV-2, we must utilise evidence from behavioural sciences in order to optimise policy adherence and create an environment which enables and motivates that behaviour.
- The second COVID-19 wave in Australia has been controlled. Daily cases at end of July were: 723 in Australia, 846 in United Kingdom, and 1,377 in France. Mid-October it was 11 in Australia, 16,171 in UK and 25,068 in France. Australian's public health response succeeded by having the right balance of government support and regulation—including a very strict and well-tolerated regional stage-4 lockdown.

## What Are the New Findings?

- We have self-reported Australian behaviours, knowledge, motivations, and concerns around COVID-19 at two important time points; after the first COVID-19 wave and at the beginning of a second COVID-19 wave.
- Strategies to support behavioural adherence with policies to limit SARS-CoV-2 spread included daily joint communication from political and public health leaders, supportive economic measures (e.g., financial income support schemes) and public health strategies (e.g., free universally accessible testing and healthcare).
- Most Australians were adherent but a small majority, mainly men and young adults, did not adequately socially distance, quarantine or test if unwell. In a geographically isolated second COVID-19 wave, additional strict lockdown and punitive measures were generally supported, adhered to and were successful.

## What Do the New Findings Imply?

- Australia has now successfully reduced COVID-19 cases from two waves of significant SARS-CoV-2 transmission, and the world might benefit from the strategies applied.
- Behavioural research has a key role to play in assisting government and informing the public on evidence based strategies in the fight against COVID-19 moving forward.

## Strengths and Limitations

- This research captured a large, representative sample of the adult Australian population across age, sex, location, and socioeconomic status.
- We have self-reported Australian behaviours, knowledge, adherence to health measures, types of concerns, and adherence motivators around COVID-19 at two important time points; after the first wave and at the beginning of a second wave, which was then successfully controlled and lessons are applicable globally.
- The survey is based on established behavioural theories, and is the Australian arm of the international iCARE survey which to date has collected global comparative information from over 90,000 respondents in 140 countries.
- Our survey was only available in English, which may have led to an underrepresentation of ethnic groups, and participation was voluntary, so our sample may be prone to selection bias from those with more interest or engagement in COVID-19.
- We also rely on self-reported behaviour, which may have led to socially desirable traits being over reported.

## Introduction

The COVID-19 pandemic has had an unprecedented impact of the lives of people around the world (1). Australia has had experience with two waves of COVID-19 and reducing COVID-19 cases successfully twice (2). Without effective treatment or a vaccine, governments globally have been forced to implement extraordinary public health measures to control the spread of disease and prevent significant loss of life. These interventions require substantial and sustained behaviour change, and come at significant personal, psychosocial and economic costs (1). If we are to be successful in containing SARS-CoV-2, we must utilise evidence from behavioural sciences in order to optimise policy adherence (3), and create an environment which enables and motivates that behaviour (4).

Health behaviour models such as the “capability,” “opportunity,” “motivation,” and “behaviour” model COM-B and the Health Beliefs Model highlight important factors influencing behaviour (3, 5–7). Examples of these factors include: perceiving a personal threat; believing a behaviour is effective to avoid that threat; possessing the capability to enact the behaviour; and having an environment which enables that behaviour. These factors may vary greatly across demographic subgroups, leading to differing patterns of behaviour (8). However, we currently have limited empirical data for use in the application of these models to COVID-19.

This study aims to understand the drivers of behaviour around COVID-19, in order to better inform public health policies. To do this we analysed two rounds of representative data (2 months apart: early May and early July 2020) consisting of self-reported behaviours, knowledge, motivations and concerns around COVID-19 by Australians. The first survey round was 1 month after the first wave peak when Australia had successfully reduced daily cases from a peak at 469 to 14 (2). The second survey round was at the start of wave 2 when daily cases had increased to 86 (and later reached a peak at 701 ~1 month later, mostly in the state of Victoria, before being successfully reduced) (2). Supplementary Table 1 shows the public health policies implemented in Australia around these times. Although the second COVID-19 wave was predominantly localised within Victoria, at the time of the second survey all other Australian states and territories were on high alert anticipating the potential rise in local cases.

This paper focuses on three key behavioural interventions designed to limit the spread, including: physical distancing; getting tested when symptoms develop; and self-quarantining. We also examine the demographics, concerns and motivators of subgroups, which are defined by varying levels of policy adherence. In doing so we aim to provide insights into policy strategies that will equip the public with the opportunity, motivation and capability (5) to adhere to key behaviours needed to control COVID-19.

## Methods

Recruitment for this longitudinal Australian survey occurred in two rounds: a first survey round, May 1st to 5th, 2020, and a second survey round, July 1st to 7th, 2020. Representative national sampling using an online survey based on evidence-based behaviour change models was conducted. Representative sampling for key demographics of the Australian population was done by sex, age, and residential location (see further below for more detail).

This project is the Australian arm of the international iCARE (International COVID-19 Awareness and Responses Evaluation) study looking at people's understanding, attitudes, beliefs and actions towards COVID-19 (coronavirus/novel coronavirus) which has to date collected over 70,000 surveys from 140 countries (8). The iCARE study is a multi-round cross-sectional observational study of people's awareness, attitudes, and responses to the COVID-19 pandemic that is tagged to national policy and case data. The study is led by the Montreal Behavioural Medicine Centre (MBMC: www.mbmc-cmcm.ca) in collaboration with a team of international collaborators. It has received REB approval from the Comité d'éthique de la recherche du CIUSSS-NIM (Centre intégré universitaire de santé et de services sociaux du Nord-de-l'île-de-Montréal), approval #: 2020-2099 / 25-03-2020. The international survey recruitment began on March 27th, 2020 (8). The Australian version of the survey is identical to the international version with the addition of four extra questions relevant to the Australian context. The project below was considered by the Monash University Human Research Ethics Committee and the committee was satisfied that the proposal meets the requirements of the National Statement on Ethical Conduct in Human Research and has granted approval (MUHREC Project ID: 24449).

### Patient and Public Involvement

As part of the main iCARE study, there are a number of community collaborators who provided input into the development of the survey design, ensuring that the items are relevant and appropriately worded, this is particularly critical when developing a survey that has to be distributed across multiple countries around the globe. To ensure the survey was applicable and relevant to the Australian population, the international iCARE survey was reviewed by the Monash Partners Consumer and Carer group prior to the first round. This involved two members paid for their time to identify text that wasn't clear or irrelevant to Australia, and recommend alternative wording and areas to clarify. Other community members and contacts of the researchers provided input into the timing to complete the survey, and subsequently this feedback resulted in the survey being shortened to reduce participant burden.

### Participants and Sampling Strategy

The first survey round with two reminders recruited 1,005 people. The 2nd survey round, along with and two reminders when needed, was sent to these 1,005 participants, yielding 648 repeat responses. New participants were then invited in another two rounds, ensuring representativeness was maintained, with an end total of 1,051 round two survey respondents.

This sample was captured by contracting an external cross-panel market research provider to send invitations to complete the online survey to ~12,000 people, using a well-established database and reimbursement in accordance with ISO 26362 and industry requirements. Reimbursement was delivered by post to a physical address, enhancing validation of respondents and avoiding limitations of other panels that reward *via* electronic means (increased numbers of professional respondents, duplication within the panel and panellists that reside outside of Australia). Participants aged 18 years and over, who resided in Australia were invited to complete the online study *via* targeted emails describing the content and estimated duration of survey. Participants were consented online, after reading the study purpose. To ensure broad representativeness, demographics of the targeted sample were aligned with the Australian Bureau of Statistics (ABS) population characteristics (9). A representative sample is a subset of a larger group and represents the same properties and proportion of a larger population. Whilst this cannot be representative across all population characteristics, it is a widely accepted approach (10) and we aimed for this sample to be consistent with the population proportions across sex, age, and residential location (state/territory and remoteness area) (Table 1). After 4 days of recruitment, age, sex, and broad location of residence (state/rurality) of participants were examined, and further sampling was targeted to underrepresented groups to align with population characteristics.

**Table 1:** Demographics of the participants who completed a round 1 survey (*n* = 1,005) and round 2 survey (*n* = 1,051) in Australia.

	**Australian population**	**Round 1**		**Round 2**		**Total**	
	**(%)**	***n***	**(%)**	***n***	**(%)**	***n***	**(%)**
**Total surveys**	-	1,005	(100)	1,051	(100)	2,056	(100)
**Sex^s^**							
Male	50	498	(49.6)	537	(51.1)	1,035	(50.3)
Female	50	499	(49.7)	507	(48.2)	1,006	(48.9)
Other	0	4	(0.4)	4	(0.4)	8	(0.4)
Missing	-	4	(0.4)	3	(0.2)	7	(0.3)
**Age^s^**							
18–29	22	90	(9.0)	266	(25.3)	356	(17.3)
30–39	18	192	(19.2)	168	(16.0)	360	(17.5)
40–49	16	161	(16.1)	154	(14.7)	315	(15.3)
50–59	16	202	(20.2)	165	(15.7)	367	(17.9)
60–69	14	191	(19.1)	132	(12.6)	323	(15.7)
70+	17	168	(16.7)	166	(15.8)	334	(16.2)
Missing	-	1	(0.1)	0	(0)	1	(0)
**Location by state/territory^s^**							
NSW	31	287	(28.6)	285	(27.1)	572	(27.8)
VIC	25	302	(30.1)	399	(38.0)	701	(34.1)
QLD	20	205	(20.4)	194	(18.5)	399	(19.4)
SA	8	88	(8.8)	85	(8.1)	173	(8.4)
WA	10	93	(9.3)	73	(7.0)	166	(8.1)
TAS	2	16	(1.6)	13	(1.2)	29	(1.4)
ACT	2	8	(0.8)	0	(0)	8	(0.4)
NT	1	0	(0)	0	(0)	0	(0)
Missing	-	6	(0.6)	0	(0)	6	(0.3)
**Location by remoteness area^s^**							
Major Cities of Australia	72	775	(77.1)	835	(79.5)	1,610	(78.3)
Inner Regional Australia	18	156	(15.5)	152	(14.5)	308	(15.0)
Outer Regional Australia	8.2	60	(6.0)	57	(5.4)	117	(5.7)
Remote/very remote Australia	1.9	10	(1.0)	5	(0.5)	15	(0.7)
Missing	-	4	(0.4)	2	(0.2)	6	(0.3)
**Income**							
Top third	30	72	(7.2)	105	(10.0)	177	(8.6)
Middle third	30	435	(43.3)	456	(43.4)	891	(43.3)
Bottom third	30	356	(35.4)	304	(28.9)	660	(32.1)
Missing	-	142	(14.1)	186	(17.7)	328	(16.0)
**Education**							
University/Postgraduate degree	52	609	(60.6)	185	(17.6)	795	(38.7)
TAFE		*Not asked*		270	(25.7)	*na*	*na*
Secondary/high school	45	364	(36.2)	250	(23.8)	614	(29.9)
Primary school or less	3	19	(1.9)	20	(1.9)	39	(1.9)
Missing	-	13	(1.3)	326	(31.0)	339	(16.5)
**Living with other adults (18 years and over)**							
No other adults		134	(13.3)	119	(11.3)	253	(12.3)
1 adult		354	(35.2)	349	(33.2)	703	(34.2)
2 adults		362	(36.0)	371	(35.2)	733	(35.7)
3 or more adults		150	(14.9)	191	(18.2)	341	(16.6)
Missing		5	(0.5)	21	(2.0)	26	(1.3)
**Living with children (under 18 years)**							
No children		731	(72.7)	768	(73.1)	1499	(72.9)
1–2 children		224	(22.3)	214	(20.4)	438	(21.3)
3 or more children		41	(4.1)	42	(4.0)	83	(4.0)
Missing		9	(0.9)	27	(2.6)	36	(1.8)
**Primary job sector before COVID-19**				233	(22.5)		
Professional				92	(8.9)		
Manager				44	(4.3)		
Technician or associate professional				111	(10.7)		
Clerical support worker				87	(8.4)		
Service and sales worker				5	(0.5)		
Skilled agricultural, forestry, and fishery worker				21	(2.0)		
Craft and related trades worker				14	(1.4)		
Plant and machine operator and assembler				17	(1.6)		
Elementary occupations				2	(0.2)		
Armed forces occupations				243	(23.5)		
Other				167	(16.1)		
Missing				233	(22.5)		

*Response rate for new participants was 10% and 63% for repeated surveys (participants who did surveys in both rounds). Participant ages ranged between 18 and 99 years, with median age of 49 years (IQR: 34–64)*.

s *Representative sampling for key demographics of Australian population was done by sex, age, and residential location. Overall, there were n = 2,056 study surveys completed*.

### Setting

Postcodes were provided by survey participants and mapped to the Australian Bureau of Statistics (ABS) remoteness areas using ABS data cubes (11, 12). Postcodes were coded by socioeconomic index for areas (SEIFA) (11, 12). Specifically, the index of relative socioeconomic disadvantage (IRSD) was applied and divided into five quintiles, from 1 (most disadvantaged) to 5 (most advantaged).

### Data Analysis

Data screening ensured data usability and an integrity script allowed discarding of surveys with <10% completion. Descriptive statistics were calculated for key survey variables for each of the two rounds of surveys. Regression analyses are described below. Multicollinearity was tested by examining the variance inflation factor (VIF) of all the variables included in the regression models. Any variable whose VIF values were >5 were further investigated for multicollinearity (13).

#### Longitudinal Survey Analyses

Mixed effects regression analyses were done with the individual specified as random effects to account for repeated measures. Mixed effects ordinal logistic regressions were then applied to examine characteristics around likelihood of adhering to three key behavioural interventions designed to limit the spread: (1) physical distancing; (2) self-quarantining; and (3) getting tested when symptoms develop. The dependent variable was likelihood of adhering to the behaviour (4 = extremely likely; 3 = somewhat likely; 2 = unlikely, and 1 = very-unlikely) and treated as ordinal as it has a natural ordering. Ordinal logistic regression requires the proportional odds assumption to be met and this was assessed using a likelihood-ratio test of whether the coefficients are equal across categories [using *omodel* and *brant, detail* (14)]. When indicated, output from the ordinal logistic regressions are displayed as proportional odds ratios. Independent variables specified as fixed effects included: sex; age-group; area IRSD; state; rurality; and education. Initial mixed effects regressions examined these independent variables, and those with *p* <0.2 were included in the final multivariate mixed effects regression.

#### Additional Analyses With the Cross-Sectional Second Survey

The second survey had additional questions, which enabled profiling of participants in regard to adherence of the three public health behaviours promoted in Australia to limit SARS-CoV-2 spread. These questions captured the likelihood of adhering to: (1) physical distancing; (2) self-quarantining; and (3) getting tested when symptoms develop. Using this information, adherence behaviour is displayed in a Sanskey diagram (15). Then profiles of people with varying adherence were created using these questions. The “adherent in all three measures” group were the participants who responded “most-of-the-time” in all three questions. The “non-adherent in at least one of the three measures” group were those who didn't respond “most-of-the-time” in all three questions. The “non-adherent in all three measures” group were those not responding “most-of-the-time” in any of the three questions. The “non-adherence of ‘never”' group were those who responded “never” to the three measures.

## Results

### Participants

The first survey round was completed by *n* = 1,005 participants and the second survey round by 1,051. Overall 2,056 surveys were completed in both survey rounds 1 and 2, and 63% (1,294/2,056) were longitudinal with 647 individuals completing both surveys. Another 762 individuals completed one of the rounds of the survey. The response rate was 10% overall and was 63% in those providing longitudinal data.

Ages ranged from 18 to 99 years (median 53, IQR: 34–64). Key demographics are shown in Table 1. Table 1 also shows expected proportions in the general Australian population. The sample obtained captured a large, representative sample of the adult Australian population across age, sex, location, and socioeconomic status.

#### Main Findings From the Longitudinal Surveys

Overall, we found the reported knowledge of Australian policies was generally high, see Supplementary Table 2.

Table 2 show the reported policy adherence on behaviours the government or health agencies recommended in response to the COVID-19 pandemic, and the degree that each has been adopted as reported by these survey participants in Australia. It shows that the majority of Australians reported being adherent “most of the time” for all policy recommended behaviours. Adherence on public health policy regarding physical distancing, self-quarantining and getting a test for COVID-19 are detailed below, and profiles of those who are likely (or not) to report adherence are also provided.

**Table 2:** Policy adherence on behaviours the government or health agencies recommended in response to the COVID-19 pandemic, and the degree that each has been adopted as reported by participants in Australia.

	**Round 1 (*****n*** **=** **1,005)**										**Round 2 (*****n*** **=** **1,056)**									
	**Total**		**Most of the time**		**Some of the time**		**Seldom**		**Never**		**Total**		**Most of the time**		**Some of the time**		**Seldom**		**Never**	
	***n***	**(%)**	***n***	**(%)**	***N***	**(%)**	***n***	**(%)**	***n***	**(%)**	***n***	**(%)**	***n***	**(%)**	***n***	**(%)**	***n***	**(%)**	***n***	**(%)**
Hand washing with soap and water for 20 s	992	(100.0)	773	(77.9)	163	(16.4)	40	(4.0)	16	(1.6)	1,034	(100)	749	(72.4)	209	(20.2)	57	(5.5)	19	(1.8)
Using hand sanitizer	980	(100.0)	460	(46.9)	326	(33.3)	141	(14.4)	53	(5.4)	1,031	(100)	562	(54.5)	344	(33.4)	92	(8.9)	33	(3.2)
Coughing/sneezing into your elbow	947	(100.0)	666	(70.3)	169	(17.8)	59	(6.2)	53	(5.6)	1,005	(100)	731	(72.7)	170	(16.9)	67	(6.7)	37	(3.7)
Wearing a face mask every time you go out of your home^***^	947	(100.0)	107	(11.3)	79	(8.3)	126	(13.3)	635	(67.1)	1,009	(100)	83	(8.2)	109	(10.8)	160	(15.9)	657	(65.1)
Wearing a face mask on public transport or crowded areas^***^	-		-		-		-		-		727	(100)	115	(15.8)	73	(10.0)	89	(12.2)	450	(61.9)
Staying at least 1.5–2 m away from other people	971	(100.0)	729	(75.1)	169	(17.4)	42	(4.3)	31	(3.2)	1,031	(100)	734	(71.2)	213	(20.7)	63	(6.1)	21	(2.0)
Staying/working at home rather than going to work or school	703	(100.0)	471	(67.0)	92	(13.1)	47	(6.7)	93	(13.2)	678	(100)	344	(50.7)	126	(18.6)	57	(8.4)	151	(22.3)
Avoiding getting take-out food or delivery	877	(100.0)	289	(33.0)	181	(20.6)	183	(20.9)	224	(25.5)	902	(100)	230	(25.5)	169	(18.7)	199	(22.1)	304	(33.7)
Avoiding all social gatherings (large and small)	953	(100.0)	768	(80.6)	105	(11.0)	43	(4.5)	37	(3.9)	959	(100)	460	(48.0)	271	(28.3)	129	(13.5)	99	(10.3)
Avoiding any non-essential travel	931	(100.0)	734	(78.8)	123	(13.2)	47	(5.0)	27	(2.9)	944	(100)	618	(65.5)	174	(18.4)	81	(8.6)	71	(7.5)
Avoiding using public transportation (except essential service workers)	678	(100.0)	499	(73.6)	79	(11.7)	46	(6.8)	54	(8.0)	594	(100)	355	(59.8)	121	(20.4)	65	(10.9)	53	(8.9)
Limiting public transport use to allow for physical distancing	-		-		-		-		-		595	(100)	342	(57.5)	114	(19.2)	63	(10.6)	76	(12.8)
Self-quarantining if you have or believe you have the virus	395	(100.0)	301	(76.2)	43	(10.9)	24	(6.1)	27	(6.8)	373	(100)	271	(72.7)	39	(10.5)	39	(10.5)	24	(6.4)
Self-isolating if you have been in contact for over 15 min with others who are awaiting test results	-		-		-		-		-		343	(100)	220	(64.1)	51	(14.9)	35	(10.2)	37	(10.8)
Self-quarantine at home if you have symptoms and are awaiting a COVID-19 result	-		-		-		-		-		416	(100)	296	(71.2)	60	(14.4)	29	(7.0)	31	(7.5)
Self-quarantine if you have had close contact with a confirmed case	-		-		-		-		-		379	(100)	248	(65.4)	63	(16.6)	33	(8.7)	35	(9.2)
Having a test as soon as you have symptoms	-		-		-		-		-	-	397	(100)	258	(65.1)	60	(15.1)	37	(9.3)	42	(10.6)
See a doctor or seek a test if you have symptoms	-		-		-		-		-	-	469	(100)	293	(62.5)	75	(16.0)	47	(10.0)	54	(11.5)

*Round 1 surveys were completed during May 1st to 5th, 2020; and round 2 surveys completed during July 1st to 7th, 2020*.

*** *Not government policy in Australia at that time*.

### Physical Distancing

**Figure 1:**
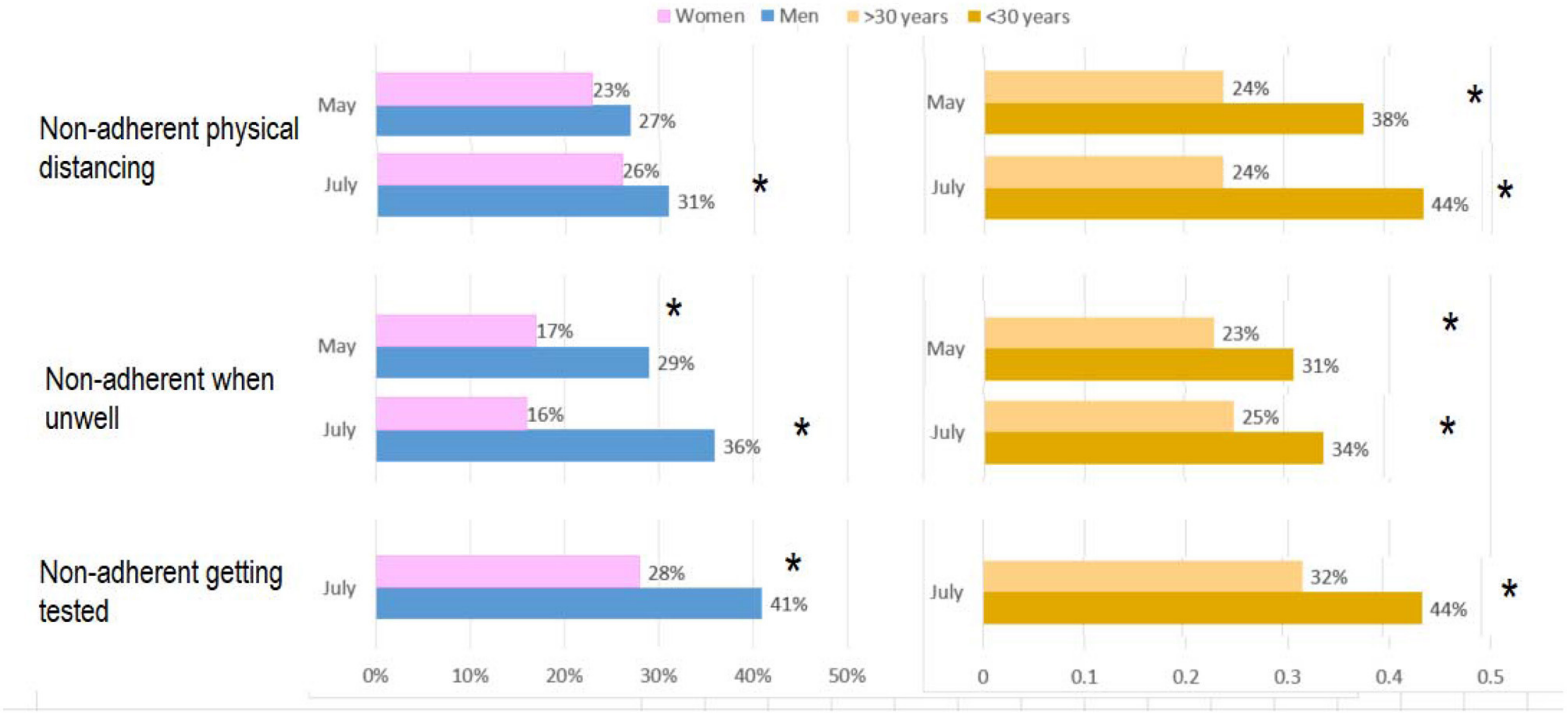
Proportion of Australians in May (round 1) and July (round 2) reporting non-adherence to physical distancing (at least 1.5 m away from other people), self-isolation when unwell (you have or think you have the virus) and having a test as soon as you have symptoms. Most-of-the-time responses provided to the question on: physical distancing was 73.1% (95% CI: 71.1–75.0); self-quarantining when unwell was 74.3% (95% CI: 72.4–76.1), and; having a test as soon as you have symptoms was 65.0% (95% CI: 60.1–69.7). *Significant difference between groups (*p* <0.05).

Not physically distancing most-of-the-time was reported in over 1-in-4 in both survey rounds with differences by sex and age: For round 1, 27% men vs. 23% women (*p* > 0.05) and 38% under 30 years vs. 19% >30 years (*p* <0.001); and for round 2, 31% men vs. 26% women (*p* = 0.04) and 46% under 30 years vs. 31% >30 years (*p* <0.001), see Figure 1 and Table 2. Mixed effects multivariate regression confirmed that after adjusting for time, age-group, location and education level, women had higher odds for physical distancing (odds ratio 1.75, 95% confidence interval 1.25–2.45) compared to men, see Table 3A. Generally, older age groups displayed higher odds for physical distancing. For example, compared to those aged 18–29 years: the odds ratio for those 40–49 years was 3.27 (1.88–5.67); 50–59 years was 4.31 (2.49–7.45); 60–69 years was 8.33 (4.54–15.30), and; 70 years and over was 8.69 (4.60–16.40).

**Table 3A:** Regression analyses: Physical distancing by staying at least 1.5–2 m away from other people.

**Outcome**	**Independent variable**		***N***	**Univariate**				**Multivariate^B^**			
				**OR**	**95% C**		***p*-value**	**OR**	**95% C**		***p*-value**
					**Lower**	**Upper**			**Lower**	**Upper**	
Physical distancing	Time	Round 1	971	1.18	0.91	1.52	0.21				
		Round 2	1,031	(ref)	-	-	-				
	Gender	Men	1,008	(ref)	-	-	-	(ref)	-	-	-
		Women	980	1.56	1.10	2.20	0.01*	1.75	1.25	2.45	<0.01*
	Age group	18–29 yo	344	(ref)	-	-	-	(ref)	-	-	-
		30–39 yo	343	0.90	0.55	1.49	0.69	0.89	0.54	1.47	0.56
		40–49 yo	305	3.29	1.89	5.71	<0.01*	3.27	1.88	5.67	<0.01*
		50–59 yo	361	4.30	2.49	7.43	<0.01*	4.31	2.49	7.45	<0.01*
		60–69 yo	319	8.44	4.64	15.32	<0.01*	8.33	4.54	15.30	<0.01*
		70 yo and over	329	8.53	4.60	15.83	<0.01*	8.69	4.60	16.40	<0.01*
	IRSD quintile	(poorest) 1	309	(ref)	-	-	-				
		2	373	1.17	0.77	1.54	0.62				
		3	428	1.12	0.71	1.38	0.97				
		4	404	0.83	0.54	1.15	0.26				
		(richest) 5	483	1.05	0.71	1.35	0.89				
	Major Cities	Other	435	(ref)	-	-	-	(ref)	-	-	-
		Major Cities	1,567	0.61	0.39	0.93	0.02*	1.03	0.69	1.56	0.94
	Major States	NSW	556	(ref)	-	-	-				
		VIC	680	1.06	0.81	1.34	0.74				
		QLD	391	0.91	0.70	1.24	0.64				
		Other	375	1.38	0.86	2.34	0.32				
	Highest edu	Secondary or less	629	(ref)	-	-	-	(ref)	-	-	-
		College / University	1,337	0.72	0.49	1.04	0.07	1.08	0.77	1.53	0.68

*Outcome has categories of: 4 = most of time; 3 = some of time; 2 = seldom, and; 1 = never*.

B *Independent variables in the multivariate model are: sex, age-group, rurality, and highest education*.

### Self-Quarantining

Figure 1 shows that in both rounds, sub-optimal policy adherence was evident including not self-quarantining most-of-the-time in 1-in-4 when unwell with large differences by sex: For round 1, 29% men vs. 17% women (*p* <0.01); and round 2, 36% men vs. 16% women (*p* <0.001). There also appeared to be some differences by age: for round 1, 31% under 30 years vs. 23% >30 years (*p* = 0.12); and round 2, 34% under 30 years vs. 25% >30 years (*p* = 0.04). Mixed effects multivariate regression showed no differences between rounds 1 and 2 when examining the self-quarantine results. Mixed effects regression showed women more likely to self-quarantine compared to men when unwell (odd ratio 3.35; 1.87–5.99), see Table 3B. Generally, older age groups displayed higher odds for self-quarantining. For example, compared to those aged 18–29 years: the odds ratio for those 50–59 years was 3.20 (1.29–7.95), and; 70 years and over was 6.10 (2.09–17.84).

### Testing

Figure 1 shows that having a test as soon as you have symptoms (only asked in the second survey round) was also sub-optimal: 59% men vs. 72% women (*p* <0.001) and 56% under 30 years vs. 68% >30 years (*p* = 0.01). Similar results were reported for seeing a doctor or seeking a test if you have symptoms: 54% men vs. 73% women (*p* <0.001) and 50% under 30 years vs. 66% >30 years (*p* = 0.001). Mixed effects multivariate regression showed that after adjusting for time, age-group and education level, women had higher odds, compared to men, see a doctor or seek a test if symptomatic (2.25; 1.51–3.45), see Table 3C. As with the physical distancing and self-quarantining results reported above, there was a general trend for higher odds ratios in the older age groups (compared to the youngest group of 18–29 years) and this was significant for those 60–69 years with 3.09 (1.44–6.61), and; 70 years and over with OR 3.58 (1.81–7.07).

### Multicollinearity

In all the regressions mentioned below, all independent variables had VIFs that were <1, indicating minimal multicollinearity.

#### Main Findings Arising From the Additional Questions in the Round Two Survey

##### Profiles of Adherence

**Figure 2:**
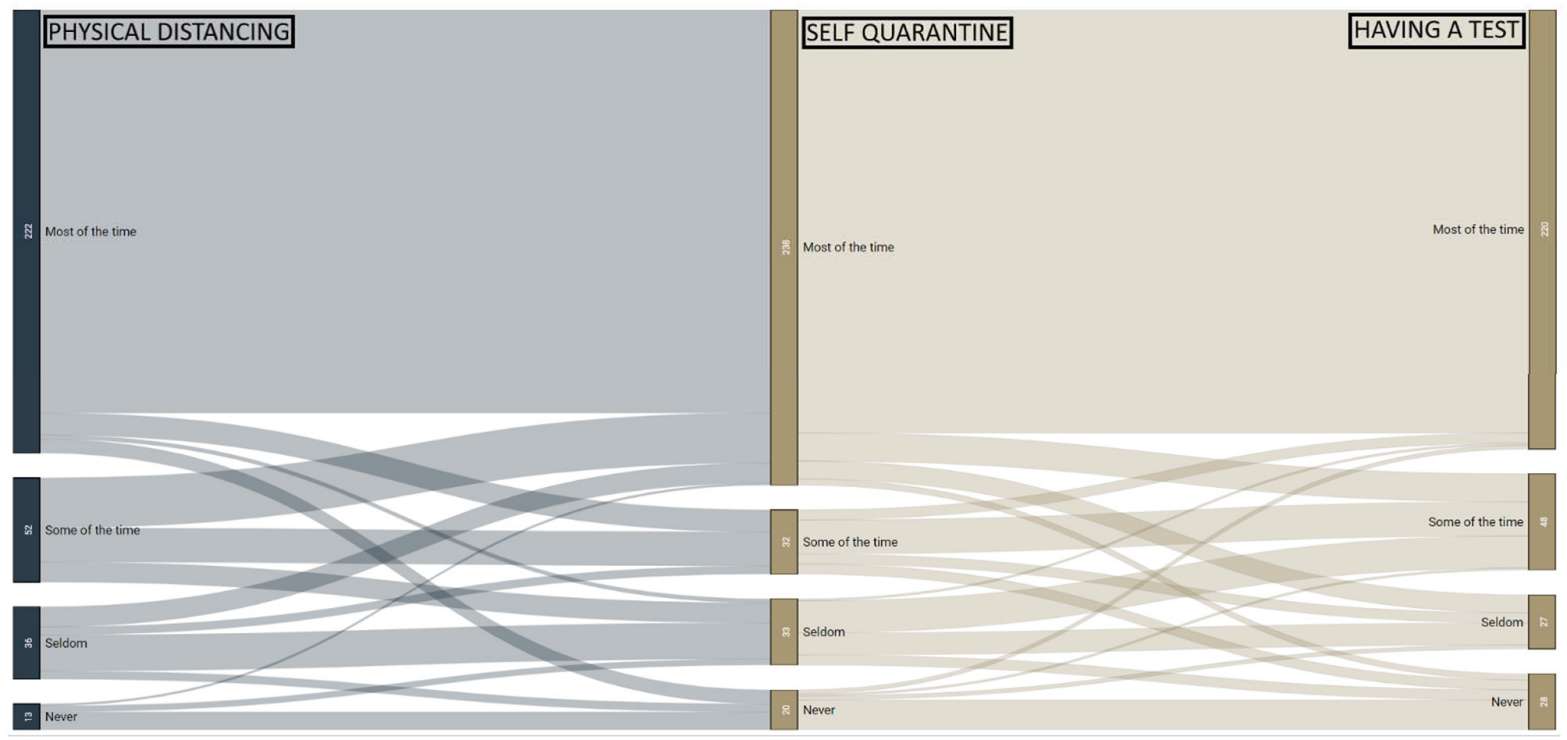
Responses to three key public health measure on physical distancing, self-quarantining, and getting tested. These three measures were available from *n* = 323 participants in round 2. Those “adherent in all three measures” (57%; 185/323) responded “most-of-the-time” in all three questions. “Non-adherent in at least one measure” were 43% (138/323). “Non-adherent in all three measures” were 19% (60/323). A small group responded “never” in each question (2.5%; 8/323).

In the second round of the survey, 323 participants responded to all three key public health measure items: physical distancing, self-quarantining and getting tested, see Figure 2. The remaining participants responded “not applicable” to at least one of these questions and were excluded for the subgroup analyses. Subgroups were then generated across levels of adherence. There were 57% (185/323) in the “adherent in all three measures” group, and 43% (138/323) in the “non-adherent in at least one of the three measures” group. There were 19% (60/323) in the “non-adherent in all three measures” group. There were 2.5% (8/323) in the “non-adherence of ‘never”' group. Supplementary Table 4 shows the demographics of these groups.

**Table 3B:** Regression analyses: Self-quarantining if you are unwell (i.e., if you have or believe you have the virus).

**Outcome**	**Independent variable**		***N***	**Univariate**				**Multivariate^B^**	
				**OR**	**95% C**		***p*-value**	**OR**	**95% C**		***p*-value**
					**Lower**	**Upper**			**Lower**	**Upper**	
Self-quarantining if you have or believe you have the virus	Time	Round 1	395	1.41	0.88	2.21	0.13	1.26	0.81	1.95	0.31
		Round 2	373	(ref)	-	-	-	(ref)	-	-	-
	Gender	Men	416	(ref)	-	-	-	(ref)	-	-	-
		Women	347	3.61	1.97	6.63	<0.01*	3.35	1.87	5.99	<0.01*
	Age group	18–29 yo	145	(ref)	-	-	-	(ref)	-	-	-
		30–39 yo	161	0.49	0.23	1.03	0.07	0.50	0.24	1.06	0.07
		40–49 yo	117	1.45	0.63	3.33	0.38	1.37	0.59	3.17	0.47
		50–59 yo	126	3.16	1.29	7.72	<0.01	3.20	1.29	7.95	0.01*
		60–69 yo	106	2.74	1.11	6.74	<0.03	2.25	0.91	5.60	0.08
		70 yo and over	113	6.55	2.31	18.60	<0.01*	6.10	2.09	17.84	<0.01*
	IRSD quintile	(poorest) 1	120	(ref)	-	-	-				
		2	146	0.98	0.39	2.46	0.78				
		3	160	1.20	0.49	2.96	0.97				
		4	145	0.86	0.76	2.17	0.42				
		(richest) 5	196	1.59	0.32	3.91	0.53				
	Major Cities	Other	140	(ref)	-	-	-	(ref)	-	-	-
		Major Cities	628	0.52	0.25	1.11	0.09	0.84	0.40	1.73	0.62
	Major States	NSW	230	(ref)	-	-	-				
		VIC	271	1.01	0.51	2.02	0.80				
		QLD	130	0.80	0.35	1.85	0.70				
		other	137	0.88	0.39	2.02	0.90				
	Highest edu	Secondary or less	226	(ref)	-	-	-				
		College / University	525	0.69	0.39	1.23	0.21				

*Outcome has categories of: 4 = most of time; 3 = some of time; 2 = seldom, and; 1 = never*.

B *Independent variables in the multivariate model are: survey round, sex, age-group, and rurality*.

**Table 3C:** Regression analyses: Having a test as soon as you have symptoms (top) and see a doctor or seek a test if you have symptoms (bottom).

**Outcome**	**Independent variable**		***N***	**Univariate**				**Multivariate^B^**			
				**OR**	**95% CI**		***p*-value**	**OR**	**95% CI**		***p*-value**
					**Lower**	**Upper**			**Upper**	**Lower**	
Having a test as soon as you have symptoms	Gender	Men	209	(ref)	-	-	-	(ref)	-	-	-
		Women	185	2.40	0.98	5.95	0.06	1.76	1.13	2.73	<0.01*
	Age group	18–29 yo	99	(ref)	-	-	-	(ref)	-	-	-
		30–39 yo	82	0.55	0.32	0.94	0.03*	0.54	0.31	0.92	0.03*
		40–49 yo	62	1.73	0.89	3.37	0.11	1.65	0.83	3.27	0.16
		50–59 yo	56	2.14	1.04	4.40	0.04*	2.08	1.00	4.30	0.05
		60–69 yo	41	2.62	1.11	6.17	0.03*	2.17	0.91	5.22	0.08
		70 yo and over	57	3.56	1.59	7.94	<0.01*	3.37	1.47	7.60	<0.01*
	IRSD quintile	(poorest) 1	59	(ref)			-				
		2	73	0.75	0.30	1.92	0.54				
		3	83	1.01	0.41	2.46	0.99				
		4	87	1.02	0.42	2.47	0.99				
		(richest) 5	95	1.15	0.47	2.78	0.82				
	Major Cities	Other	73	(ref)	-	-	-	(ref)	-	-	-
		Major Cities	324	0.66	0.38	1.14	0.14	0.89	0.46	1.73	0.73
	Major States	NSW	98	(ref)	-	-	-				
		VIC	166	1.14	0.69	1.88	0.61				
		QLD	70	1.04	0.56	1.92	0.90				
		other	36	1.38	0.70	2.71	0.35				
	Highest edu	Secondary or less	105	(ref)	-	-	-				
		College / University	278	0.89	0.55	1.42	0.62				
See a doctor or seek a test if you have symptoms	Gender	Men	264	(ref)	-	-	-				
		Women	202	2.28	1.55	3.56	<0.01*	2.25	1.51	3.45	<0.01*
	Age group	18–29 yo	111	(ref)	-	-	-	(ref)	-	-	-
		30–39 yo	91	0.85	0.51	1.41	0.52	0.89	0.55	1.51	0.69
		40–49 yo	71	1.76	0.97	3.21	0.06	1.75	0.94	3.24	0.08
		50–59 yo	67	1.42	0.79	2.57	0.24	1.46	0.80	2.67	0.21
		60–69 yo	53	3.14	1.49	6.63	<0.01*	3.09	1.44	6.61	<0.01*
		70 yo and over	76	3.30	1.70	6.40	<0.01*	3.58	1.81	7.07	<0.01*
	IRSD quintile	(poorest) 1	81	(ref)			-	(ref)			-
		2	96	1.62	0.77	2.49	0.29	1.58	0.84	2.96	0.14
		3	83	2.33	0.94	3.25	0.12	1.72	0.85	3.45	0.10
		4	96	1.63	0.75	2.38	0.29	1.48	0.79	2.76	0.20
		(richest) 5	113	2.58	1.03	3.24	0.08	1.93	0.97	3.84	0.05
	Major Cities	Other	95	(ref)	-	-	-				
		Major Cities	374	0.81	0.51	1.29	0.38				
	Major States	NSW	117	(ref)	-	-	-				
		VIC	182	1.05	0.66	1.66	0.83				
		QLD	88	0.92	0.53	1.58	0.76				
		other	82	1.09	0.61	1.93	0.78				
	Highest edu	Secondary or less	115	(ref)	-	-	-				
		College / University	339	0.90	0.58	1.40	0.65				

*Only asked in the second survey round. Outcome categories of: 4 = most of time; 3 = some of time; 2 = seldom, and; 1 = never*.

B *Independent variables in the multivariate models are those with outputs shown*.

In the “adherent in all three measures” group, 54% (99/185) were women and 70% (129/185) were aged 40 years and over (Supplementary Table 3A). In the people who indicated “non-adherence in at least one of the three measures,” 64% (88/138) were men and 65% (42/138) were aged under 40 years (Supplementary Table 3B). In the “non-adherent in all three measures,” 75% (45/60) were men and 70% (42/60) were aged under 40 years (Table 3C). In those who responded “never” in each question, all (8/8) were men and 63% (5/8) were aged under 40 years, and the remaining 37% (3/8) were aged between 40 and 59 years (Supplementary Table 3D).

Supplementary Table 5 shows the concerns reported by the above adherent profile groups, and the overall round 2 participants (*n* = 1,051). Those in any of the “non-adherent” groups reported much lower concerns than those in the “adherent” group.

Supplementary Table 6 shows the things that would convince participants to practice social/physical isolation or distancing reported by round 2 participants (*n* = 1,051) and by the above adherent profile groups. Those “non-adherent in all three measures” reported very few strategies that would motivate them to adhere.

Reported attitudes on importance (Supplementary Table 7A) and severity (Supplementary Table 7B) of government measures to reduce COVID-19 spread, show that 81% (835/1,032) considered these “very important.” In those adherent in all three questions, 91% (164/181) considered these “very important, compared with 22% (13/59) in those who indicated “non-adherent in all three measures.” Overall 75% (772/1,029) considered the severity of government measures “about right,” while only 5% (51/1,029) considered government measures “too strict.” In those who indicated adherence to all three questions, only 1.6% (3/183) considered measures “too strict,” compared with 22% (13/60) among those in the “non-adherent in all three measures” group.

Participants were also asked to list barriers to having a COVID-19 test (Supplementary Table 8). The most commonly cited responses were “having to request your contacts to isolate” (21%), “having to isolate until results return (20%), “don't know where to get a test” (15%), and “inconvenience of obtaining a test” (15%). Compared to those who were adherent in all three key behaviours, those who were non-adherent in all three behaviours were more likely to be deterred by “inconvenience in obtaining a test” (22 vs. 12%).

##### Information Sources

**Figure 3:**
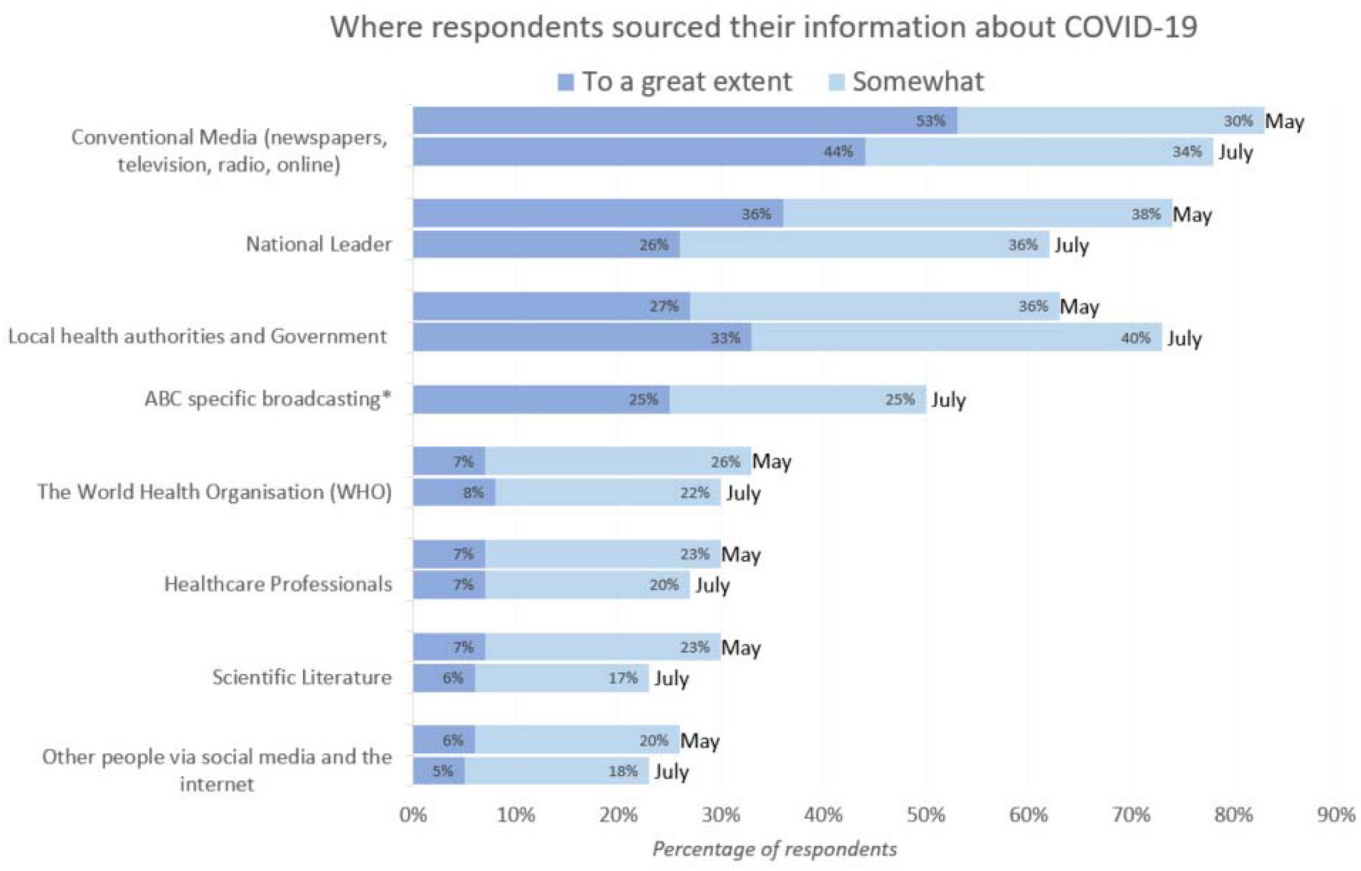
Where Australians get their information (*n* = 2,056). *Not asked in round 1 (May 2020). The majority reported sourcing information from conventional media, and this included ABC specific broadcasting for 50%.

Figure 3 shows the majority of participants sourced information from conventional media sources, including 50% of people utilising the public broadcaster, the Australian Broadcasting Corporation (ABC). In round 1, 74% of the population sourced information from the national leader, making it the second most popular source of information after conventional media sources. However, in round 2, “local health authorities and government” were the second most popular source of information, being utilised by 73% of respondents. Young people were more likely to report using other media sources such as social media.

## Discussion

The COVID-19 pandemic continues to have major health, psychosocial and economic impacts (1, 16), with Australia having successfully reduced cases following two waves, the second one focused in one state (Victoria) (2). Public health policies and behavioural change remain our primary defence (4). Here we report COVID-19 related attitudes, knowledge, concerns, and behaviours in Australia after suppression of the first wave (survey round 1) and 2 months later at the beginning of the second wave (survey round 2). Timing of the second survey round is important because the COVID-19 wave 2 was subsequently localised to Victoria, yet at the time of the second survey all areas in Australia were anxiously anticipating potential increases in cases, which might account for why our survey data didn't produce differential results when broken down by state. Overall, we found that knowledge of Australian policies was generally high, yet one in four reported non-adherence to physical distancing and self-quarantining, and one in three to testing when unwell. This likely accounts for the community transmission causing wave 2 following cases contracted from returned travellers in quarantine at city hotels especially in Victoria (2, 17). Understanding those for which adherence was lower, is crucial for designing additional and future strategies, and as seen globally, in Australia this was males and young people. Those who were non-adherent to all three policy measures reported fewer COVID-19 related concerns and were less likely to respond to behavioural motivators such as education and to regulatory, punitive measures. Overall interpretation from the Australian data is consistent with the evidence on what to do to promote adherence during pandemics (18) that education, consistent messaging, addressing concerns and motivators and providing support are all critical for behaviour change (3, 5–7), which then controlled the second wave. However, given persistent limited adherence largely in young adults and men, a geographically focused second wave required lockdown restrictions with threats of punitive outcomes. In the context of consistent communication, financial support and free healthcare, restrictive and punitive measures were generally supported and accepted as not overly restrictive and the second wave was controlled.

Behavioural and social sciences provide vital insights to enable and support behaviour change (1, 4, 19, 20). Behavioural adherence to viral spread prevention policies, can be understood through established behaviour change theories such as capability, opportunity and motivation (COM-B) and the Health Beliefs model (HBM) (3, 5–7). Examples from the data on those that are less adherent that are consistent with these models included: greater concerns of not having enough money for food and rent (capability [COM-B]) or accessing COVID-19 testing [perceived barriers (HBM), opportunity (COM-B)]; lower perceived risk from COVID-19 [perceived susceptibility (HBM), motivation (COM-B)]; and lower perceived importance of government measures [perceived severity (HBM), motivation (COM-B)]. These aspects can be leveraged to develop appropriate intervention plans for future potential waves of COVID-19 infections.

Behaviour is underpinned by knowledge, attitudes and beliefs (3, 5–7). To change behaviour, policies need to influence knowledge, attitudes and beliefs and provide the opportunity, capability and motivation to change. Diverse policy approaches include education, incentives and enablers and regulation focused on physical distancing, isolating when unwell or diagnosed and testing if unwell, all vital in slowing the spread of COVID-19. In most countries, including Australia, these policy approaches have been largely successful through consistent messaging, addressing concerns and motivators, and providing support for behaviour change (see Supplementary Table 2). However, during a crisis such as the beginning of the second wave, these were not been sustainably achieved through individual behaviour alone. In the state of Victoria, border control, regulated strict extended lock downs and punitive measures were needed, in addition to vigorous contact tracing and testing. Additional extensive policy measures implemented included: individual and business financial support, free universally accessible testing and healthcare, reimbursement to cover testing time and sick leave for self-quarantine. Over 70% supported government policies and felt they were appropriate with very few public protests (21). The second COVID-19 wave was later successfully controlled bringing locally acquired cases down from 701/day to 28/day in 6 weeks, and to zero in another 6 weeks (2).

To prevent further waves and lockdowns, optimising behavioural policy adherence is critical.

Regarding physical distancing policy adherence, we report that one in four Australians, especially young adults and men, do not adequately adhere to relevant policies, aligned with other COVID-19 (22, 23), and prior pandemic studies (24). Modelling suggests that 90% of the Australian population needs to physically distance, whilst viral control is not possible with 70% adherence, even with concomitant case isolation (25). Here, knowledge on physical distancing policies were high; however, attitudes and beliefs were problematic in young adults and young men, with non-adherers endorsing few of the concerns and motivators to change within the survey, highlighting the potential need for regulatory measures in a subgroup of the population. This aligns with the reported strong community support for government policies and general support for the severity of current government regulations in Australia (21, 26). Perceptions of a lack of policy importance or undue severity were most prominent among those who did not adhere to physical distancing, self-quarantine and testing if unwell, which is consistent with the reported lack of concerns around COVID-19.

Self-quarantine when unwell or COVID-19 positive, when returning from overseas travel, after potential contact with a known case, or if testing positive, is crucial to contain COVID-19 (27). While it is intuitive to quarantine when unwell, it remains equally important in other scenarios, particularly as we learn more about significant levels of pre-symptomatic (28) and asymptomatic (29) transmission. Even limited non-adherence can lead to widespread community transmission, as seen through reports of “super-spreaders” (30, 31). Indeed Australia's outbreak appears, on genomic testing, to have come from a single family of four returning from overseas (32). In light of this, reported high-levels of non-adherence to self-quarantining are very concerning. Despite being aware of policy recommendations and being concerned about infecting others, nearly a quarter of older people and a third of younger people and men, reported non-adherence to self-quarantining in both rounds of the survey. This is consistent with recent data collected by the Australian Defence Force through door-knocking, with reports that one in four people with confirmed infection, were not at home despite being instructed to quarantine (33). The factors underpinning quarantining non-adherence are likely complex and multifactorial. Motivation to quarantine may be affected by a poor risk perception (34), optimism bias (35), and high levels of concern in young people about being socially isolated. Furthermore, individuals may have reduced capability to self-quarantine due to the economic costs, and fears over job security (36). In order to mitigate any financial barriers to self-quarantining and testing, policy makers have since introduced a $1,500 payment to those who test positive, and $300 for Victorians who can't work while they're awaiting test results. This policy is supported by research from Israel suggesting that financial compensation can significantly improve COVID-19 quarantine adherence (37).

With regards to testing behaviours, in the midst of the July second COVID-19 wave, one third of our respondents were non-adherent to testing at the onset of symptoms. A concurrent, non-representative survey found non-adherence to be as high as 55% (38). Our findings appear consistent with reports that 30% of international travellers refused to be tested in Australia's quarantine hotels (39). Testing is vital in case identification, quarantine and contact tracing, especially as COVID-19 can be asymptomatic (40). On average, each person has around 9 close contacts (41). With every day that an individual delays getting tested after developing symptoms, the number of contacts for tracing and viral spread increases. A recent study found that a delay in testing of 3 days or more, would render even the most efficient contract tracing regimes unable to bring the R0 (effective reproduction number) below one (40). There is evidence that contact tracing in Victoria was overwhelmed, a key driver for the government to implement stage 4 lockdown (42). There are many factors that may underpin suboptimal testing adherence. Testing is free for everyone in Australia, residents and non-residents, so cost was not a factor. Among our participants, the most commonly cited concern about getting tested was “having to ask contacts to self-isolate.” This may point to the role of social stigma (43) adversely impacting motivation to be tested, as has been reported across Asia (44). Similarly, individuals' capability to be tested may be reduced by poor communication (45) about testing locations and inconvenience of getting to a test site. Sites for testing were increased over time, now with over 190 across the state of Victoria, and are a mix of pop up sites in high risk areas, walk-in, drive through, primary care, pathology, and hospital based with wait times available online. Additionally, a more recent option is “call-to-test” where in home testing is provided for those experiencing injury, health, mobility or other issues that impede their ability to leave home or their careers. At times in identified high risk areas, there was also door to door testing offered. Whilst testing is free to all residents and non-residents there are financial burdens associated with missing work and self-quarantining particularly for young people, who are more likely to be part of the casual workforce without access to sick leave (36). Hence government policies implemented in the second COVID-19 wave, which provided financial compensation for individuals/parents/carers of those getting tested and staying home, were probably important. This included (Aus $450) for testing and staying home whilst waiting for a result, as well as funding pandemic leave at normal pay for 2 weeks for those not able to access sick leave (e.g., casual workers, self -employed). Pandemic leave was available for a number of situations including: symptoms consistent with COVID-19, and isolating as a close contact of a suspected/known case of COVID-19.

To guide targeted policy interventions, we analysed survey responses by varying levels of adherence to our three key behaviours; physical distancing, self-quarantining and getting tested. Those who were non-adherent to all three measures, were more likely to be younger, male and liver in major cities. These trends are consistent with behavioural data from previous pandemics (24) and may speak to the role of risk perception in enacting behaviour change, as theorised in the Health Beliefs model (5). For example, during the SARS outbreak in 2003, women and older people were more likely to perceive themselves as high risk, and also more likely to adhere to behavioural policies such a quarantine (34). Similarly, our data shows that those who were non-adherent across all three behaviours reported significantly lower concerns around COVID-19, were more likely to consider government measures ineffective or too “strict,” than those who were adherent across all three behaviours. To increase adherence in this group, local health authorities and government should focus on emphasising the risks and consequences of contracting SARS-CoV-2. Those who were non-adherent in all three behaviours, were also far less responsive to common motivators of policy adherence (e.g., education and punitive measures). The exception to this, was a significant proportion of non-adherent individuals who reported that they would be motivated by information showing how their actions saved lives. Interestingly, research from the United Kingdom and Germany has found that inducing empathy for society's most vulnerable increases behavioural adherence during the COVID-19 pandemic, and presents a potential strategy for government messaging to a group which is otherwise difficult to target (46). Potentially engaging role models, emphasising social norms (the majority who do adhere) may be useful (1); however, these findings also emphasise that for the small majority, government regulation and punitive measures remain important (1). This is akin to public health approaches such as in smoking, and driving behaviours which involve education, incentives and regulation.

The interpretation from the Australian data is that education, consistent messaging, addressing concerns and motivators and providing support are all critical for behaviour change. However, in the context of an escalation in cases, as in the Australian second COVID-19 wave, restrictions with threats of punitive outcomes, are likely to have a role and that if these occur in the context of widespread education, consistent targeted messaging, financial support for the vulnerable populations, then they are reasonably well-tolerated, seen to be fair and not overly restrictive.

### Strengths and Limitations

This research captured a large, representative sample of the adult Australian population across age, sex, location, and socioeconomic status. The survey questionnaire was based on established behavioural theories and we were able to compare findings at two separate time points. As the Australian arm of the international iCARE survey, our data can be subsequently compared with other countries. Our survey was only available in English, which will have led to an underrepresentation of ethnic groups. There is also no data on subgroups such as single mothers, at higher risk of economic and psychosocial stressors. As the survey was voluntary, our sample may be prone to selection bias. We also rely on self-reported behaviour, which may have led to socially desirable traits being over reported (social desirability bias) (47); however, this may be mitigated by the anonymity of survey responses. Only 323 completed the provided responses to three key public health measures on physical distancing, self-quarantining and getting tested as for many, self-quarantine, and testing had not been indicated.

## Conclusion

Australia is emerging from a second wave of COVID-19, with Victoria worst affected and currently in stage four lockdown. This nationally representative survey examined adherence to three key behaviours critical to limiting the spread of COVID-19, as key targets for health policy. In both May and July 2020, adherence to key policies was suboptimal; one in four reported being non-adherent to physical distancing and self-quarantining and one in three people reported non-adherence to getting tested when unwell. Modelling suggests that these levels of adherence are inadequate to contain SARS-CoV-2, in the absence of lockdown conditions and these must be effectively addressed if further waves are to be avoided. Despite the majority of the population being adherent to public health behaviour changes, those who were non-adherent to all three policies were more likely to be male, younger and live in major cities. Sub-optimal adherence in young people and males is likely driven by poor risk perception and an inadequate concerns and beliefs in the importance of government policy, necessary to overcome the psychosocial, and economic costs of adherence. Communication strategies should focus on emphasising the personal risks of contracting COVID-19, and evoking empathy for society's most vulnerable. Support strategies need to minimise inconvenience and costs of policy adherence. Finally, sustained payments for those in quarantine and getting tested may be useful to remove barriers to adherence in groups that are financially vulnerable. Where these policies fail, poor risk perception and adherence will need to be mitigated through government punitive measures such as regulation and fines. Overall, our research emphasises the need to change community to behaviour to avoid further lockdowns and associated physical, social, and economic costs.

## Data Availability Statement

Data can be made available to approved researchers by contacting the corresponding author.

## Ethics Statement

The studies involving human participants were reviewed and approved by Monash University Human Research Ethics Committee (MUHREC Project ID: 24449). The patients/participants provided their written informed consent to participate in this study.

## iCARE Team

Lead investigators: Kim L. Lavoie, PhD, University of Quebec at Montreal (UQAM) and CIUSSS-NIM, CANADA; Simon L. Bacon, PhD, Concordia University and CIUSSS-NIM, CANADA.Collaborators (in alphabetical order): AUSTRALIA: Jacqueline Boyle, PhD, Monash University; Joanne Enticott, PhD, Monash University; Shajedur Rahman Shawon, PhD, Centre for Big Data Research in Health, UNSW Medicine; Helena Teede, MD, Monash University; AUSTRIA: Alexandra Kautzky-Willer, MD, Medizinische Universität Wien; BANGLADESH: Arobindu Dash, MS, International University of Business, Agriculture & Technology; BRAZIL: Marilia Estevam Cornelio, PhD, University of Campinas; Marlus Karsten, Universidade do Estado de Santa Catarina - UDESC; Darlan Lauricio Matte, PhD, Universidade do Estado de Santa Catarina - UDESC; CANADA:Shawn Aaron, PhD, Ottawa Hospital Research Institute; Tracie Barnett, PhD, McGill University;Silvana Barone, MD, University of Montreal; Ariane Belanger-Gravel, PhD, Université Laval; Sarah Bernard, PhD, Université Laval; Lisa Maureen Birch, PhD, Université Laval; Susan Bondy, PhD, University of Toronto - Dalla Lana School of Public Health; Linda Booij, PhD, Concordia University; Roxane Borgès Da Silva, PhD, University of Montreal; Jean Bourbeau, MD, McGill University; Rachel Burns, PhD, Carleton University; Tavis Campbell, PhD, University of Calgary; Linda Carlson, PhD, University of Calgary; Kim Corace, PhD, University of Version: 2020-09-09.

Ottawa; Olivier Drouin, MD, CHU Sainte-Justine/Université de Montréal; Francine Ducharme, MD, University of Montreal; Mohsen Farhadloo, Concordia University; Richard Fleet MD, PhD, Université Laval; Gary Garber, MD, University of Ottawa/Public Health Ontario; Lise Gauvin, PhD, University of Montreal; Jennifer Gordon, PhD, University of Regina; Roland Grad, MD, McGill University; Samir Gupta, MD, University of Toronto; Kim Hellemans, PhD, Carleton University; Catherine Herba PhD, UQAM; Heungsun Hwang, PhD, McGill University; Lisa Kakinami, PhD, Concordia University; Sunmee Kim, PhD, University of Manitoba; Sandra Pelaez, PhD, University of Montreal; Louise Pilote, MD, McGill University; Paul Poirier, MD, Université Laval; Justin Presseau, PhD, University of Ottawa; Eli Puterman, PhD, University of British Columbia; Joshua Rash, PhD, Memorial University; Paula AB Ribeiro, PhD, MBMC; Mohsen Sadatsafavi, PhD, University of British Columbia; Paramita Saha Chaudhuri, PhD, McGill University; Jovana Stojanovic, PhD, Concordia University; Eva Suarthana, MD, PhD, University of Montreal / McGill University; Michael Vallis, PhD, Dalhousie University; CHILE: Nicolás Bronfman Caceres, PhD, Universidad Andrés Bello; Manuel Ortiz, PhD, Universidad de La Frontera; Paula Beatriz Repetto, PhD, Universidad Católica de Chile; COLOMBIA: Mariantonia Lemos-Hoyos, PhD, Universidad EAFIT; CYPRUS: Angelos Kassianos, PhD, University of Cyprus; DENMARK: Naja Hulvej Rod, PhD, University of Copenhagen; FRANCE: Mathieu Beraneck, PhD, Université de Paris; CNRS; Greg Ninot, PhD, University of Montpellier; GERMANY: Beate Ditzen, PhD, Heidelberg University; Thomas Kubiak, PhD, Mainz University; GHANA: Sam Codjoe MPhil,MSc, University of Ghana; Lily Kpobi, PhD, University of Ghana; Amos Laar, PhD, University of Ghana; INDIA: Sylvia Fernandez Rao, PhD, Indian Council of Medical Research; Naorem Kiranmala Devi, PhD, University of Delhi; Suzanne Tanya Nethan, MDS, ICMR-National Institute of Cancer Prevention & Research; Lancelot Pinto, MD, PhD, Hinduja Hospital and Medical Research Centre; Kallur Nava Saraswathy, PhD, University of Delhi; Dheeraj Tumu, MD, World Health Organization (WHO); INDONESIA: Silviana Lestari, MD, PhD, Universitas Indonesia; Grace Wangge, MD, PhD, SEAMEO Regional Center for Food and Nutrition; IRELAND: Molly Byrne, PhD, National University of Ireland, Galway; Jennifer McSharry, PhD, National Universityof Ireland, Galway; Oonagh Meade, PhD, National University of Ireland, Galway; Gerry Molloy, PhD, National University of Ireland, Galway; Chris Noone, PhD, National University of Ireland, Galway; ISRAEL: Hagai Levine, MD, Hebrew University; Anat Zaidman-Zait, PhD, Tel-Aviv University; ITALY: Stefania Boccia, PhD, Università Cattolica del Sacro Cuore; Ilda Hoxhaj, MD, Università Cattolica del Sacro Cuore, Valeria Raparelli, PhD, Sapienza - University of Rome; Drieda Zaçe, MD, MSc, PhDc, Università Cattolica del Sacro Cuore; JORDAN: Ala'S Aburub, PhD, Isra University; KENYA: Daniel Akunga, PhD, Kenyatta University; Richard Ayah, PhD, University of Nairobi, School Public Health; Chris Barasa, MPH, University of Nairobi, School Public Health; Pamela Miloya Godia, PhD, University of Nairobi; Elizabeth W. Kimani-Murage, PhD, African Population and Health Research Center; Nicholas Mutuku, PhD, University of Kenya; Teresa Mwoma, PhD, Kenyatta University; Violet Naanyu, PhD, Moi University; Jackim Nyamari, PhD, Kenyatta University; Hildah Oburu, PhD, Kenyatta University; Joyce Olenja, PhD, University of Nairobi; Dismas Ongore, PhD, University of Nairobi; Abdhalah Ziraba, PhD, African Population and Health Research Center; LITHUANIA: Emeljanovas Arunas, PhD, Vilnius University; Natalja Fatkulina, PhD, Vilnius University; Brigita Mieziene, PhD, Vilnius University; MALAWI: Chiwoza Bandawe, PhD, University of Malawi; MALAYSIA: Loh Siew Yim, PhD, Faculty of medicine, University of Malaya; NEW ZEALAND: Boyd Swinburn, MD, University of Auckland; NIGERIA: Ademola Ajuwon, PhD, University of Ibadan; PAKISTAN: Nisar Ahmed Shar, PhD, CoPI-National Center in Big Data & Cloud Computing; Bilal Ahmed Usmani, PhD, NED University of Engineering and Technology; PERU: Rosario Mercedes Bartolini Martínez, PhD, Instituto de Investigacion Nutricional; Hilary Creed-Kanashiro, M.Phil., Instituto de InvestigacionNutricional; PORTUGAL: Paula Simão, MD, S. Pneumologia de Matosinhos; RWANDA: Pierre Claver Rutayisire, PhD, University Rwanda; SAUDI ARABIA: Abu Zeeshan Bari, PhD, Taibah University; SLOVAKIA: Iveta Nagyova, PhD, PJ Safarik University - UPJS; SOUTH AFRICA: Jason Bantjes, PhD, University of Stellenbosch; Brendon Barnes, PhD, University of Johannesburg; Bronwyne Coetzee, PhD, University of Stellenbosch; Ashraf Khagee, PhD, University of Stellenbosch; Tebogo Mothiba, PhD, University of Limpopo; Rizwana Roomaney, PhD, University of Stellenbosch; Leslie Swartz, PhD University of Stellenbosch; SWEDEN: Anne Berman, PhD, Karolinska Institutet; Nouha Saleh Stattin, MD, Karolinska Institutet; SWITZERLAND: Susanne Fischer, PhD, Version: 2020-09-09.

University of Zurich; TAIWAN: Debbie Hu, MD, MSc, Tainan Municipal Hospital; TURKEY: Yasin Kara, MD, KanuniSultan Süleyman Training and Research Hospital, Istanbul; Ceprail Simşek, MD Health Science University; Bilge Üzmezoglu, MD, University of Health Science; UGANDA: John Bosco Isunju, PhD, Makerere University School of Public Health; James Mugisha, PhD, University of Uganda; UK: Lucie Byrne-Davis, PhD, University of Manchester; Paula Griffiths, PhD, Loughborough University; Joanne Hart, PhD, University of Manchester; Will Johnson, PhD, Loughborough University; Susan Michie, PhD, University College London; Nicola Paine, PhD, Loughborough University; Emily Petherick, PhD, Loughborough University; Lauren Sherar, PhD, Loughborough University; USA: Robert M. Bilder, PhD, ABPP-CN, University of California, Los Angeles; Matthew Burg, PhD, Yale; Susan Czajkowski, PhD, NIH - National Cancer Institute; Ken Freedland, PhD, Washington University; SherriSheinfeld Gorin, PhD, University of Michigan; Alison Holman, PhD, University of California, Irvine; Gilberto Lopez ScD, MA, MPH, Arizona State University and University of Rochester Medical Center; Sylvie Naar, PhD, Florida State University; Michele Okun, PhD, University of Colorado, Colorado Springs; Lynda Powell, PhD, Rush University; Sarah Pressman, PhD, University of California, Irvine; Tracey Revenson, PhD, University of New York City; John Ruiz, PhD, University of Arizona; Sudha Sivaram, PhD, NIH, Center for Global Health; Johannes Thrul, PhD, Johns Hopkins; Claudia Trudel-Fitzgerald, PhD, Harvard T.H. Chan School of Public Health.Students (in alphabetical order): AUSTRALIA: Rhea Navani, BSc, Monash University; Kushnan Ranakombu, PhD,Monash University; BRAZIL: Daisuke Hayashi Neto, Unicamp; CANADA: Anda Dragomir, University of Quebec at Montreal (UQAM) and CIUSSS-NIM; Amandine Gagnon-Hébert, BA, UQAM; Claudia Gemme, MSc, UQAM; Vincent Gosselin Boucher, University of Quebec at Montreal (UQAM) and CIUSSS-NIM; Mahrukh Jamil, Concordia University and CIUSSS-NIM; Lisa Maria Käfer, McGill University; Tasfia Tasbih, Concordia University and CIUSSS-NIM; Maegan Trottier, University of Lethbridge; Ariany Marques Vieira, MSc, Concordia University; Robbie Woods, MSc, Concordia University; Reyhaneh Yousefi, Concordia University and CIUSSS-NIM; FRANCE: Tamila Roslyakova, University Montpellier; GERMANY: Lilli Priesterroth,Mainz University; ISRAEL: Shirly Edelstein, Hebrew University-Hadassah School of Public Health; Tanya Goldfrad, Hebrew University-Hadassah School of Public Health; Ruth Snir, Hebrew University-Hadassah School of Public Health; Yifat Uri, Hebrew University-Hadassah School of Public Health; NEW ZEALAND: Mohsen Alyami, University of Auckland; NIGERIA: Comfort Sanuade; SERBIA: Katarina Vojvodic, University of Belgrade;Community Participants: CANADA: Olivia Crescenzi; Kyle Warkentin; DENMARK: Katya Grinko; INDIA: Lalita Angne; Kulka Bharati, MD; Jigisha Jain; Nikita Mathur, Syncorp Clinical Research; Anagha Mithe; Sarah Nethan, Community Empowerment Lab. Funding: iCARE was supported by the Canadian Institutes of Health Research (CIHR: SMC-151518), Fonds de recherche du Québec - santé (FRQ-S: 251618 and 34757), and the Fonds de recherche du Québec – Société et culture (FRQSC: 2019-SE1-252541). Study sponsors had no role in the design of the database and data collection.

## Author Contributions

SB and KL led study conceptualisation. JE was responsible for the statistical aspects analyses. JE and WS wrote the first draft of the paper. JB and HT are the senior authors and guarantors. All authors contributed to the development of the research question, study design in relation to the Australian data analysis, interpretation of the results, critical revision of the manuscript for important intellectual content, and approved the final version of the manuscript. JB attests that all listed authors meet authorship criteria and that no others meeting the criteria have been omitted.

## Conflict of Interest

The authors declare that the research was conducted in the absence of any commercial or financial relationships that could be construed as a potential conflict of interest.

## References

[1] Bavel J Baicker K Boggio PS Capraro V Cichoka A Cikara M. Using social and behavioural science to support COVID-19 pandemic response. Nat Hum Behav. (2020) 4:460–71. 10.1038/s41562-020-0884-z 32355299

[2] Government A. Coronavirus (COVID-19) current situation and case numbers Canberra, Australia. (2020). Available online at: https://www.health.gov.au/news/health-alerts/novel-coronavirus-2019-ncov-health-alert/coronavirus-covid-19-current-situation-and-case-numbers#daily-reported-cases (accessed December 6, 2020).

[3] West R Michie S Rubin G Amlot R. Applying principles of behaviour change to reduce SARS-CoV-2 transmission. Nat Hum Behav. (2020) 4:451–9. 10.1038/s41562-020-0887-9 32377018

[4] Michie S West R. Behavioural, environmental, social, and systems interventions against covid-19. BMJ. (2020) 370:m2982. 10.1136/bmj.m2982 32723732

[5] Michie S West R Campbell R Brown J Gainforth H. ABC of Behaviour Change Theories. Surrey: Silverback Publishing (2014).

[6] Rosenstock I. The health belief model and preventive health behavior. Health Educ Monogr. (1974) 2:354–86. 10.1177/109019817400200405

[7] Michie S van Stralen M West R. The behavior change wheel: a new method for characterizing and designing behavior change interventions. Implement Sci. (2011) 6:42. 10.1186/1748-5908-6-42 21513547 PMC3096582

[8] Bacon SL Lavoie KL Boyle J Stojanovic J Joyal-Desmarais K. An international assessment of the link between COVID-19-related attitudes, concerns and behaviours in relation to public health policies: optimising policy strategies to improve health, economic and quality of life outcomes (the iCARE Study): protocol paper. BMJ Open. (2021) 11:e046127. 10.1136/bmjopen-2020-046127 PMC7956731 33707274

[9] Statistics ABo. Australian Demographics Statistics 2020. cat. no. 3101.0. (2020). Available online at: https://www.abs.gov.au/Ausstats/abs@.nsf/0/41FC8AB241938C05CA258479001A763E?OpenDocument (accessed December 6, 2020).

[10] Webb P Bain C Page A. Essential Epidemiology: An Introduction for Students and Health Professionals. 3rd ed. Cambridge: Cambridge University Press (2017). p. 494.

[11] Statistics ABo. Australian Statistical Geography Standard. (2018). Available online at: https://www.abs.gov.au/AUSSTATS/abs@.nsf/DetailsPage/1270.0.55.005July%202016?OpenDocument (accessed December 6, 2020).

[12] Welfare AIoHa. Rural & Remote Health Canberra: AIHW. (2019). Available online at: https://www.aihw.gov.au/getmedia/918ae1b7-eeaf-4e64-9b88-36b887100a9e/Rural-remote-health.pdf.aspx?inline=true (accessed December 6, 2020).

[13] Sheather SJ. A modern approach to regression with R introduction. Springer Texts Stat. (2009) 2009:1. 10.1007/978-0-387-09608-7_1

[14] Wolfe R Gould W. An approximate likelihood-ratio test for ordinal response models. Stata Technical Bulletin 42: 24–27 In Stata Technical Bulletin Reprints, vol 7. College Statio, TX: Stata Press (1998). p. 199–204.

[15] Charts G. Sankey Diagram UTC. (2020). Available online at: https://developers.google.com/chart/interactive/docs/gallery/sankey#top_of_page (accessed December 6, 2020).

[16] Siegfrid A. Global Cost of Coronavirus May Reach $4.1 Trillion, ADB Says bloomberg.com. (2020). Available online at: https://www.bloomberg.com/news/articles/2020-04-03/global-cost-of-coronavirus-could-reach-4-1-trillion-adb-says (accessed December 6, 2020).

[17] Coate J. Board of Inquiry Into the COVID-19 Hotel Quarantine Program. Final Report Victoria, Australia (2020). Available online at: https://www.quarantineinquiry.vic.gov.au/covid-19-hotel-quarantine-inquiry-final-report-0 (accessed December 5, 2020).

[18] Bonell C Michie S Reicher S West R Bear L Yardley L. Harnessing behavioural science in public health campaigns to maintain “social distancing” in response to the COVID-19 pandemic: key principles. J Epidemiol Community Health. (2020) 74:617–9. 10.1136/jech-2020-214290 32385125 PMC7368244

[19] Kulge H. Statement – Behavioural Insights Are Valuable to Inform the Planning of Appropriate Pandemic Response Measures euro.who.int. World Health Organisation (2020). Available online at: https://www.euro.who.int/en/health-topics/health-emergencies/coronavirus-covid-19/statements/statement-behavioural-insights-are-valuable-to-inform-the-planning-of-appropriate-pandemic-response-measures (accessed December 6, 2020).

[20] Smith L Curtis J. Coronavirus stage 4 rules are hard but behavioural science knows what makes you toe the line. ABC News (2020). Available online at: https://www.abc.net.au/news/2020-08-06/can-victorians-stick-to-stage-4-rules-coronavirus-restrictions/12529028 (accessed December 5, 2020).

[21] Murphy K. Essential Poll: Victorians Overwhelmingly Support Harsh Restrictions to Curb Covid Second Wave. The Guardian Essential Report (2020). Available online at: https://www.theguardian.com/australia-news/2020/aug/12/essential-poll-victorians-overwhelmingly-support-harsh-restrictions-to-curb-covid-second-wave (accessed December 5, 2020).

[22] Public perceptions of COVID-19 in Australia: perceived risk knowledge health-protective behaviours and vaccine intentions. Vaccine Weekly. 2020 2020/05/20/. 10.3389/fpsyg.2020.551004 PMC7561403 33117223

[23] BehaviourWorks. COVID-19 Scrub Survey Wave 2: What Are Australians Doing and Who Are They Listening To? Monash Sustainable Development Institute (2020). Available online at: https://www.behaviourworksaustralia.org/covid-19-scrub-survey-wave-2-what-are-australians-doing-and-who-are-they-listening-to/ (accessed December 6, 2020).

[24] Bish A Michie S. Demographic and attitudinal determinants of protective behaviours during a pandemic: a review. Br J Health Psychol. (2010) 15:797–824. 10.1348/135910710X485826 20109274 PMC7185452

[25] Chang SL Harding N Zachreson C Cliff OM Prokopenko M. Modelling transmission and control of the COVID-19 pandemic in Australia. Nat Commun. (2020) 11:5710. 10.1038/s41467-020-19393-6 33177507 PMC7659014

[26] Essential Research. Government Response to Covid-19: The Essential Report (2020). Available online at: https://essentialvision.com.au/state-government-response-to-covid-19-15 (accessed December 5, 2020).

[27] West R Michie S. Routes of transmission of SARS-CoV-2 and behaviours to block it: a summary. Qeios. (2020) 1:2. 10.32388/F6M5CB.2

[28] He X Lau EHY Wu P Deng X Wang J Hao X. Temporal dynamics in viral shedding and transmissibility of COVID-19. Nat Med. (2020) 26:672–5. 10.1038/s41591-020-0869-5 32296168

[29] Lee S Kim T Lee E Lee C Kim H Rhee H. Clinical course and molecular viral shedding among asymptomatic and symptomatic patients with SARS-CoV-2 infection in a community treatment center in the Republic of Korea. JAMA Internal Med. (2020) 2020:3862. 10.1001/jamainternmed.2020.3862 32780793 PMC7411944

[30] Cave E. COVID-19 super-spreaders: definitional quandaries and implications. Asian Bioeth Rev. (2020) 2020:1–8. 10.1007/s41649-020-00118-2 PMC7229875 32427202

[31] McGraw E. A Few Superspreaders Transmit the Majority of Coronavirus Cases. The Conversation. (2020). Available online at: https://theconversation.com/a-few-superspreaders-transmit-the-majority-of-coronavirus-cases-139950 (accessed June 5, 2020).

[32] Burton T. One Family of Four Source of 90 per cent of Australia's Second Wave. Financial Review. (2020). Available online at: https://www.afr.com/politics/federal/one-family-of-four-source-of-90-per-cent-of-australia-s-second-wave-20200818-p55mu7 (accessed August 30, 2020).

[33] Handley E. More Than a Quarter of Victoria Coronavirus Patients Not at Home When Doorknocked by ADF abc.net.au. ABC News (2020). Available online at: https://www.abc.net.au/news/2020-07-31/one-in-four-not-home-covid19-positive-adf-door-knock/12511682 (accessed December 6, 2020).

[34] Cava MA Fay KE Beanlands HJ McCay EA Wignall R. Risk perception and compliance with quarantine during the SARS outbreak. J Nurs Scholar. (2005) 37:343–7. 10.1111/j.1547-5069.2005.00059.x 16396407

[35] Park T Ju I Ohs JE Hinsley A. Optimistic bias and preventive behavioral engagement in the context of COVID-19. Res Soc Admin Pharmacy. (2020) 17:1859–66. 10.1016/j.sapharm.2020.06.004 33317765 PMC7836537

[36] Whiteford P Bradbury B. >If We Want Workers to Stay Home When Sick, We Need Paid Leave for Casuals theconversation.com: The Conversation. (2020). Available online at: https://theconversation.com/if-we-want-workers-to-stay-home-when-sick-we-need-paid-leave-for-casuals-138431 (accessed December 6, 2020).

[37] Bodas M Peleg K. Self-isolation compliance in the COVID-19 era influenced by compensation: findings from a recent survey in Israel. Health Affairs. (2020) 39:936–41. 10.1377/hlthaff.2020.00382 32271627

[38] Health Do. FluTracking Reports (Australia) info.flutracking.net. Australian Government (2020). Available online at: https://info.flutracking.net/reports-2/australia-reports/ (accessed December 6, 2020).

[39] Hayne J. States have power to keep people in hotel quarantine if they refuse coronavirus tests, CMO says. ABC News (2020). Available online at: https://www.abc.net.au/news/2020-06-26/national-cabinet-coronavirus-testing-hotel-quarantine/12396360 (accessed December 5, 2020).

[40] Kretzschmar ME Rozhnova G Bootsma MCJ van Boven M van de Wijgert J Bonten MJM. Impact of delays on effectiveness of contact tracing strategies for COVID-19: a modelling study. Lancet Public Health. (2020) 5:e452–9. 10.1016/S2468-2667(20)30157-2 32682487 PMC7365652

[41] Purtill J. “A Real Sleuthing Exercise:” Every Coronavirus Case Starts a Race to Track Contacts abc.net.au. Australian Broadcasting Comission (2020). Available online at: https://www.abc.net.au/triplej/programs/hack/every-coronavirus-case-starts-a-race-to-track-contacts/12021878 (accessed December 6, 2020).

[42] Dow A Fowler M. ‘It's dire': contact tracing delays threaten coronavirus fight. The Age (2020). Available online at: https://www.abc.net.au/news/2020-06-26/national-cabinet-coronavirus-testing-hotel-quarantine/12396360 (accessed December 5, 2020).

[43] WHO. Social Stigma Associated with COVID19 who.int. World Health Organisation (2020). Available online at: https://www.who.int/docs/default-source/coronaviruse/covid19-stigma-guide.pdf (accessed December 6, 2020).

[44] Bloomberg. Social Stigma and Harassment Undermine COVID-19 Testing Efforts Across Asia japantimes.co.jp. Bloomberg (2020). Available online at: https://www.japantimes.co.jp/news/2020/05/13/asia-pacific/stigma-harassment-coronavirus-testing-asia/ (accessed December 6, 2020).

[45] Grey A. Multilingual Australia Is Missing Out on Vital COVID-19 Information. No Wonder Local Councils and Businesses Are Stepping in theconversation.com. The Conversation (2020). Available online at: https://theconversation.com/multilingual-australia-is-missing-out-on-vital-covid-19-information-no-wonder-local-councils-and-businesses-are-stepping-in-141362 (accessed December 6, 2020).

[46] Pfattheicher S Nockur L Böhm R Sassenrath C Petersen M. The emotional path to action: empathy promotes physical distancing and wearing face masks during the COVID-19 pandemic. Psychol Sci. (2020) 31:y2cg5. 10.31234/osf.io/y2cg5 32993455

[47] Rosenman R Tennekoon V Hill LG. Measuring bias in self-reported data. Int J Behav Healthc Res. (2011) 2:320–32. 10.1504/IJBHR.2011.043414 25383095 PMC4224297

