# Supplementary material for: Knowledge, Attitude, and Self-Reported Practice Towards Measures for Prevention of the Spread of COVID-19 Among Australians: A Nationwide Online Longitudinal Representative Survey
Source: Front Public Health. 2021 Jun 2;9:630189. doi: 10.3389/fpubh.2021.630189 (PMC8206530; doi:10.3389/fpubh.2021.630189)
Supplement: Supplementary file 1 [file Data_Sheet_1.PDF]

Supplementary Table 1: Australian Federal, State and Territory policies and regulations in response to COVID-19*
**from February to April, Federal policies are emphasised. From April/June onwards, Victorian & NSW policies are emphasised as the second wave was localised to these states.*

Date	Policy measure	Further details
**Travel restrictions**		
01/02/2020	First ban on foreign nationals entering Australia from nominated countries.<br><br>Australian residents and citizens not allowed to travel to nominated country.<br><br>Returning travellers from nominated countries required to self-isolate at home for 14 days.	Specific dates when countries banned:<br>• 1st February: China<br>• 1st March: Iran<br>• 5th March: Korea<br>• 11th March: Italy<br>• 18th March: All Countries
15/03/2020	Return travellers from *ALL* countries required to self-isolate at home for 14 days<br><br>Ban on cruise ships from foreign ports	
18/03/2020	All outbound and in-bound travel banned<br>Australian citizens and residents told to return home or risk being unable to	Exception:<br>"essential" travel including humanitarian and medical reasons
19/03/2020	First interstate border-restrictions:<br><br>Tasmania closes borders to all "non-essential travellers" unless they undertake mandatory 14 day quarantine	Subsequent border restrictions:<br>• 24th March – Northern Territory, Western Australian and South Australia<br>• 11th April – Queensland<br>• 8th July - Victorian-NSW border closed<br><br>Subsequent easing/tightening of border restrictions between states has occurred on a discretionary basis depending on case numbers
27/03/2020	Australian Mandatory Hotel Quarantine Program Introduced	

30/06/2020	All flights to Victoria diverted to other Australian cities temporarily	
**Physical, social and event restrictions**		
16/03/2020	Ban on non-essential, organised gatherings of ≥500 people	Exceptions:<br>Schools, universities, workplaces, public transport, domestic travel and public transient places
18/03/2020	Ban on non-essential indoor gatherings of ≥ 100 people<br><br>Physical distancing of 1.5 metres introduced in all settings	Exceptions:<br>Public transport, medical/health care facilities, pharmacies, emergency services, correctional facilities, youth justice and some others
20/03/2020	For gatherings <100 people, floor space of 1 person per 4 square metres required with at least 1.5 metres distance between people	
30/03/2020	Stay at home requirements introduced	Exceptions to "stay at home requirement":<br>1. medical or healthcare needs<br>2. daily exercise<br>3. work or study if unable to complete at home<br>4. shopping for essential items
	Indoor and outdoor gatherings restricted to two people only	Each State and Territory applied this rule separately (as examples, Victoria banned all non-essential social visits, whereas Queensland later allowed two known visitors to each household)
8/5/2020	National three stage framework to lift restrictions announced	Several regions began lifting restrictions earlier;<br>• 27 April, Western Australia, allowed 10 people at some indoor and outdoor non-work gatherings<br>• 1st May, NSW allowed two adults to visit another household

15/5/2020	QLD and NT further ease restrictions in accordance with national framework	
31/5/2020	Victoria steps down from stage 3 restrictions	1) Increase to 20 people at household/outdoor gatherings; 2) Increase to 20 people at weddings or religious ceremonies, up to 50 people at a funeral; 3) Entertainment, cultural venues, swimming pools to re-open; 4) Auctions & open for inspections subject to 20 person limit; 5) Next set of easing restrictions from 22 June
22/6/2020	Victoria cancels plans to ease restrictions, tightening social measures in light of rising case numbers	Households only allowed to have five visitors at home; Outside gatherings of families and friends will be limited to 10 people
08/07/2020	Stage 3 restrictions applied to Melbourne and Mitchel shire following beginning of second wave	
10/07/2020	**Facemasks** recommended in Melbourne when not at home<br>Made compulsory in Melbourne on 22nd July<br>Recommended in Victoria on 30th July<br>Made compulsory in all Victoria on 2nd August	
02/08/2020	Tightening of restrictions to contain 2nd Wave<br>1) Victorian state of disaster declared<br>2) Stage 4 restrictions for Melbourne<br>3) Stage 3 restrictions for regional Victoria<br>4) All schools return to remote learning	2) stage 4 details: curfew announced from 8 pm to 5 am, shopping for essential items must only be done within a 5 km radius with only one person per household allowed to shop per day, exercise within five kilometres of the home for one hour maximum, once daily; outdoor gatherings limited to two people<br>4) kindergarten/childcare only open for children of vulnerable / essential workers in metropolitian Melbourne, will remain open to all in regional areas
06/09/2020	Victorian government announced roadmap for easing restrictions tied to case numbers	

**Business restrictions**		
23/03/2020	Initial restrictions on businesses set	Some non-essential venues closed, including hotels (excluding accommodation), clubs, gyms, indoor sporting venues, cinemas, entertainment venues, casinos, night clubs, religious gatherings, places of worship or funerals, except very small groups (observing previous social distancing rules).<br><br>Restaurants and cafes open for take-away or delivery only.
25/03/2020	Further restrictions on businesses set	Most non-essential venues closed, including but not limited to, beauty therapy, tanning, waxing, nail salons, tattoo parlours, spas, massage parlours, play centres, amusement parks, health clubs, museums, libraries, pools, youth centres and community facilities<br><br>Hairdressers, boot camp and personal trainings (maximum 10 people) remain open<br><br>Weddings (maximum 5 people), funerals (maximum 10 people) with social distancing
31/5/2020	Victoria eases stage three business restrictions	
14/06/2020	Victorian announces that business restrictions will ease further from the 21st of June	Plan for libraries & community halls to open up to 50 people; recommencement of sports etc
21/06/2020	Victoria eases restrictions for gyms, cinemas etc as planned, with a few exceptions	20 person limit to remain for all businesses, despite initial plan to allow 50 people
06/08/2020	Victorian business closures for at least 6 weeks across retail, administration and some manufacturing	Some stores to remain operational via delivery and “click and collect”
**Punitive measures – fines, arrest, penalties**		
16/03/2020	States begin introducing fines for:<br>• failing to self-isolate<br>• breaching event restrictions	\$11,000 - \$50,000 depending on state, possible prison sentences

27/3/2020	QLD announced fines for deliberately coughing or spitting on someone	$13,345 per person
29/03/2020	Victoria announces fines for breaches including gatherings, failure to quarantine, business regulations etc.	$1652 for non-essential travel<br>$9,913 for businesses
18/07/2020	Victoria: $200 fine for not wearing mask	
17/07/2020	New fines for venues breaching COVID safe restrictions/regulations in some states	Up to $55,000 per venue, plus $27,500 for every additional day the breach is continued
04/08/2020	Victoria announces spot fines for breaching lockdown rules	$4,957 per person
**Financial support for business**		
12/03/2020	Increased instant asset write-off (IAWO) threshold from $30, 000 to $150, 000	Government to provide up to $100, 000 to eligible small and medium-sized businesses and not-for-profits; with a minimum payment of $20,000.
22/03/2020	Increased cash flow for employers	
30/03/2020	Wage subsidy scheme introduced for eligible employers	JobKeeper payment introduced - $1500 payment per fortnight (before tax) for each eligible employee/ self-employed individuals for up to six months
**Financial support for individuals and households**		
12/03/2020	First $750 payment to eligible recipients announced, to be delivered by 13th April	Available to social security, veteran and other income support recipients and eligible concession card holders
22/03/2020	Second $750 payment to eligible recipients announced, to be delivered on 10th July	
	Coronavirus supplement announced	Eligible income support recipients receive $550 per fortnight for six months from 27/4/20 in addition to JobSeeker allowance
24/03/2020	JobSeeker and Youth Allowance income support payment eligibility expanded	Eligible recipients include newly unemployed sole traders, self-employed, casual workers, contract workers, eligible individuals caring for someone affected by coronavirus

29/03/2020	Temporary moratorium on evictions from rental properties for people facing financial distress	
22/07/2020	Victoria announces payment to assist those awaiting test results<br>• $300 whilst awaiting test results, if unable to work<br>• $1500 whilst self-isolating for two weeks, if unable to work	$300 payment increased to $450 on the 13th of August
**School and education centres**		
22/03/2020	Federal Government encourages schools to remain open until term 1 break	Each State and Territory applied this rule separately (some commenced Term 1 break early on 24th March)
06/04/2020	Families receive free childcare	
16/04/2020	Federal Government advises schools to open for Term 2, but individual states apply discretion	E.g. South Australia opened schools for Term 2, NSW and Victoria announced a staggered plan to return students with first students returning on 11th and 26th of May respectively
08/7/2020	Victoria: All year levels except year 11 and 12 delayed return to school from holidays for 1 week	
02/8/2020	Victoria: All students return to remote learning	
**Healthcare policies**		
13/03/2020	New temporary Medicare Benefits Schedule (MBS) Telehealth numbers to support free telehealth medical consultations	For consultations for individuals at risk of COVID-19 infection or those who have self-isolated as a result of COVID-19 (13th March), expanded to all Medicare-eligible[1] Australians (30th March)
18/03/2020	Aged care visitor restrictions introduced for:<br>• More than 2 people<br>• Anyone with contact with COVID-19<br>• Anyone with symptoms	Exceptions: Discretionary groups allowed for end of life situations
19/03/2020	Limited prescription medication purchasing to avoid stockpiling	

- [1] Medicare is Australia's health care that enables free or lower cost community health care and free hospital based health services. It covers Australian and New Zealand citizens, permanent residents and some others. https://www.healthdirect.gov.au/what-is-medicare

25/03/2020	Temporary suspension of all semi-urgent elective surgery in the public hospital and private hospital system (1st April).	Policy eased 27th April
29/03/2020	Funding announced for domestic violence, mental health support and for other vulnerable Australians	
09/08/2020	Victoria: $60 million for mental health support announced	
**Testing**		
10/03/2020	South Australia and Victoria open first "drive through" testing clinics<br><br>$205 Million project announced to establish 100 "pop-up" testing clinics across Australia	Other states quickly follow:<br>• NSW – 12th March<br>• QLD – 14th March<br>• ACT – 19th March<br><br>COVID-19 tests free for everyone
26/02/2020	Expanded national minimum testing criteria	Testing criteria expanded to include people with fever or acute respiratory infection in at-risk categories including, but not limited to, all health workers, all aged/residential workers, hospitalised patients with symptoms of unknown cause, geographically localised areas where there is elevated risk or high-risk settings.
24/04/2020	Expanded national minimum testing criteria to include mild symptoms	Testing to include all people with mild symptoms of COVID-19
14/06/2020	Victoria announces targeted testing strategy to end of August	Targeting local government areas with low testing rates, communities with high case numbers, high-risk workforces and vulnerable groups
25/06/2020	Victoria announces testing blitz of 10 "priority" suburbs in Melbourne	Asymptomatic testing in chosen areas

28/06/2020	1. National mandatory testing on day 3 and 11 for all returned travellers in hotel quarantine<br><br>2. Introduction of saliva sample test to increase testing uptake in prescribed circumstances	1. Those who refuse to comply are forced to remain in hotel quarantine for an additional 10 days
**Self-quarantine and self-isolation**		
15/03/2020	For all returned travellers from all countries, 14 days	
26/03/2020	For confirmed cases and those who suspect they have COVID-19 or have symptoms, 14 days	To be nationally consistent at the State and Territory level
**Contract tracing**		
26/04/2020	Voluntary tracking and tracing app released	COVIDSafe App aims to assist the Government in contact tracing, by recording close contact between two users of the app
01/06/2020	Some states introduce mandatory "check-in" details for customers in restaurants, cafes etc.	
18/09/2020	Nationally consistent approach to contact tracing announced	
