# Supplementary material for: Knowledge, Attitude, and Self-Reported Practice Towards Measures for Prevention of the Spread of COVID-19 Among Australians: A Nationwide Online Longitudinal Representative Survey
Source: Front Public Health. 2021 Jun 2;9:630189. doi: 10.3389/fpubh.2021.630189 (PMC8206530; doi:10.3389/fpubh.2021.630189)
Supplement: Supplementary file 2 [file Data_Sheet_2.PDF]

Supplementary Tables 2 to 8

*Supplementary Table 2. Reported knowledge on policies recommended by government or health agencies recommended in response to the COVID-19 pandemic. *Not applicable (NA), missing data and "I prefer not to answer" responses are combined. Empty cells indicate that the question wasn't asked in the first round.*

**Knowledge on policies and behaviours believed to be recommended by government**	**First round (n=1,005)**						**Second round (n=1,051)**					
	**NA***		**No**		**Yes**		**NA***		**No**		**Yes**	
	**n**	**(%)**	**n**	**(%)**	**n**	**(%)**	**n**	**(%)**	**n**	**(%)**	**n**	**(%)**
Staying at least 1.5 to 2 metres away from other people	30	(3.0)	154	(15.3)	821	(81.7)	15	(1.4)	36	(3.4)	1000	(95.1)
Self-quarantining if you have or believe you have the virus	34	(3.4)	52	(5.2)	919	(91.4)	35	(3.3)	50	(4.8)	966	(91.9)
Having a test as soon as you have symptoms (fever, cough, sore throat, shortness of breath, loss of smell, runny nose)	-		-		-		54	(5.1)	77	(7.3)	920	(87.5)
See a doctor or seek a test if you have symptoms (fever, cough, sore throat, shortness of breath, loss of smell, runny nose)	-		-		-		50	(4.8)	87	(8.3)	914	(87.0)
Hand washing with soap and water for 20 seconds	13	(1.3)	12	(1.2)	980	(97.5)	11	(1.0)	28	(2.7)	1012	(96.3)
Avoiding any non-essential travel	23	(2.3)	32	(3.2)	950	(94.5)	37	(3.5)	138	(13.1)	876	(83.3)
Coughing/sneezing into your elbow	22	(2.2)	45	(4.5)	938	(93.3)	11	(1.0)	36	(3.4)	1004	(95.5)
Using hand sanitizer	29	(2.9)	46	(4.6)	930	(92.5)	19	(1.8)	40	(3.8)	992	(94.4)
Staying/working at home rather than going to work or school	37	(3.7)	85	(8.5)	883	(87.9)	58	(5.5)	203	(19.3)	790	(75.2)
Coughing/sneezing into a tissue, throw it away and wash your hands	61	(6.1)	109	(10.9)	835	(83.1)	63	(6.0)	131	(12.5)	857	(81.5)
Self-quarantining if you are returning from a trip	31	(3.1)	49	(4.9)	925	(92.0)	55	(5.2)	80	(7.6)	916	(87.2)
Avoiding using public transport (except essential service workers)	100	(10.0)	315	(31.3)	590	(58.7)	88	(8.4)	407	(38.7)	556	(52.9)
Wearing a face mask every time you go out of your home**	62	(6.2)	751	(74.7)	192	(19.1)	62	(5.9)	767	(73.0)	222	(21.1)

Supplementary Table 3. The age and sex of those who provided responses to three key questions about physical distancing, self-quarantining and getting tested in survey round 2 (n=323); for adherence profile definitions see Figure 2.

(a) In those who indicated adherence in all three questions, 54% (99/185) were women and 70% (129/185) were aged 40 years and over.

Age group

	**18-29**	**30-39**	**40-49**	**50-59**	**60-69**	**70+**	**Total**
**Men**	17	9	18	17	8	16	85
	20.0	10.6	21.2	20.0	9.4	18.8	100.0
**Women**	18	12	19	16	16	18	99
	18.2	12.1	19.2	16.2	16.2	18.2	100.0
**Other**	0	0	0	0	0	1	1
	0	0	0	0	0	100	100
**Total**	35	21	37	33	24	35	185

(b) *In the people who indicated non-adherence in at least one of the three questions, 64% (88/138) were men and 65% (42/138) were aged under 40 years.*

Age group

	**18-29**	**30-39**	**40-49**	**50-59**	**60-69**	**70+**	**Total**
**Men**	28	31	8	11	2	8	88
	31.8	35.2	9.1	12.5	2.3	9.1	100.0
**Women**	14	16	8	4	6	1	49
	28.6	32.7	16.3	8.2	12.2	2.0	100.0
**Other**	0	0	1	0	0	0	1
	0	0	100	0	0	0	100
**Total**	42	47	17	15	8	9	138

(c) *In the people who indicated non-adherence in all three questions, 75% (45/60) were men and 70% (42/60) were aged under 40 years.*

Age group

	**18-29**	**30-39**	**40-49**	**50-59**	**60-69**	**70+**	**Total**
**Men**	14	18	5	6	0	2	45
	31.1	40.0	11.1	13.3		4.4	100.0
**Women**	5	5	2	1	0	1	14
	35.7	35.7	14.3	7.1		7.1	100.0
**Other**			1				1
			100				100
**Total**	19	23	8	7	0	3	60

(d) In those who responded "never" in each question, all (8/8) were men and 63% (5/8) were aged under 40 years, and the remaining 37% (3/8) were aged between 40 and 59 years.

Age group

	**18-29**	**30-39**	**40-49**	**50-59**	**60-69**	**70+**	**Total**
**Men**	1	4	2	1	0	0	8
	12.5	50.0	25.0	12.5			100.0

Supplementary Table 4. Further information about the round 2 participants (n=1051) by varying groups of adherence to the three key questions about physical distancing, self-quarantining when unwell and getting tested (for profile definitions see Figure 3).

	Adherent	Non-adherent			Total
	**Adherent in all three (n=185)**	**Non-adherence in at least one of the three (n=138)**	**Non-adherence in all three (n=60)**	**Non-adherence of "never" in all three (n=8)**	**Round 2 (n=1051)**
	**%**	**%**	**%**	**%**	**%**
**Men**	46.2	64.2	76.2	100.0	**51.4**
**Aged <40 years**	21.2	64.5	70.0	62.5	**41.3**
**Lives in a major city**	77.3	86.2	95.0	100.0	**79.5**
**Do you have any form of paid sick leave from your job? "YES"**	58.6	52.2	49.1	25.0	**54.5**
**Do you currently have private health insurance? "YES"**	56.1	56.7	50.0	37.5	**56.8**
**Healthcare worker**	6.2	19.4	18.6	25.0	**7.2**
**Essential service worker as defined by your country (healthcare, police, etc)**	17.5	26.4	23.6	25.0	**18.2**
**Prior to Covid-19, receiving unemployment insurance / benefits**	6.3	9.2	14.6	25.0	**7.8**
**Currently receiving unemployment insurance / benefits**	7.0	11.5	12.5	25.0	**9.3**
**Currently employed or working either full-time and/or part-time**	48.2	64.3	61.1	57.1	**53.8**
**Over the past 5 years, received the seasonal flu vaccine every year**	47.5	27.3	19.0	12.5	**40.1**
**Never smoked regular tobacco cigarettes**	59.4	58.8	51.7	50.0	**62.4**
**Never used vaping or electronic cigarettes products**	94.4	82.5	71.2	62.5	**95.6**
**Uses vaping or electronic cigarettes products "occasionally/daily"**	5.6	17.5	28.8	37.5	**4.4**
**Downloaded and installed the government COVID-19 tracing app (COVID safe) on your phone "YES"**	51.9	36.8	30.0	0.0	**42.4**

Supplementary Table 5. Concerns reported as "To a great extent" in round 2 participants (n=1051) and by varying profile groups of adherence to three key public health measures (for profile definitions see Figure 3).

	**Adherent**	**Non-adherent**			**Total**
	**Adherent in all three (n=185)**	**Non-adherence in at least one of the three (n=138)**	**Non-adherence in all three (n=60)**	**Non-adherence of "never" in all three (n=8)**	**Round 2 (n=1051)**
	**%**	**%**	**%**	**%**	**%**
**… being infected myself**	39.0	11.9	5.2	25.0	**22.2**
**… the impact of being infected on my health, including dying**	40.9	16.3	5.0	25.0	**26.3**
**… being isolated from other people**	31.3	11.2	6.8	12.5	**19.4**
**… losing my job / family income**	39.0	18.8	10.9	12.5	**27.7**
**… losing my personal / family savings**	37.6	14.6	12.1	12.5	**26.2**
**… not having enough money for food and/or rent**	29.4	10.8	5.3	37.5	**19.3**
**… infecting other people I live with**	52.4	19.4	5.4	25.0	**41.3**
**… infecting other people in the community**	48.0	20.6	5.3	25.0	**36.2**
**… a person I live with being infected and/or dying**	56.5	20.6	7.4	25.0	**43.8**
**… a family member I don't live with being infected and/or dying**	58.5	18.8	5.2	25.0	**41.9**
**…not being able to see my friends, socialise**	28.9	10.4	6.9	12.5	**20.3**
**…the healthcare system becoming overloaded/not being able to care for the sick**	47.8	13.5	5.3	25.0	**33.4**
**… there not being enough food left on shelves**	27.8	10.5	3.6	12.5	**15.8**
**… my country going into an economic recession/depression**	45.9	22.2	13.8	12.5	**37.5**
**… how long it will take for things to "go back to normal"**	51.1	19.1	10.9	14.3	**37.4**
**… there being a second wave of COVID-19 infections in the future**	63.9	26.5	7.3	12.5	**46.2**

Supplementary Table 6. What would convince you to practice social/physical isolation or distancing "To a great extent" as reported by round 2 participants (n=1051) and by varying groups of adherence to three public health measures (for profile definitions see Figure 3.)

	Adherent	Non-adherent			Total
	Adherent in all three (n=185)	Non-adherence in at least one of the three (n=138)	Non-adherence in all three (n=60)	Non-adherence of "never" in all three (n=8)	Round 2 (n=1051)
	%	%	%	%	%
**Threat of fines**	59.9	23.7	6.7	0.0	**47.0**
**Threat of arrest/jail**	66.3	26.5	3.5	0.0	**55.3**
**Threat of institutional quarantine (e.g., in a hospital or care centre)**	63.8	21.6	5.1	0.0	**48.9**
**Threat of recognition as the source of an outbreak**	64.8	25.2	8.5	0.0	**55.4**
**Threat of tightening restrictions**	63.3	20.0	8.6	0.0	**49.1**
**Providing information on local infection/death rates**	57.7	15.7	5.0	0.0	**44.3**
**Providing information about infection/death rates outside my country**	49.1	20.6	10.2	0.0	**30.1**
**Providing information about how COVID-19 is spread**	63.1	17.4	5.2	0.0	**45.3**
**Providing information on risk factors for COVID-19-related complications or death**	62.6	17.7	10.9	0.0	**44.6**
**Providing information on how your actions can cause a new outbreak**	65.9	21.8	6.9	0.0	**46.9**
**Providing information about having limited healthcare resources**	59.6	25.4	8.6	0.0	**43.7**
**Providing information about how your actions are slowing the spread of infection…**	62.4	24.0	7.0	0.0	**45.5**
**Providing information about how your actions are saving lives**	63.8	15.1	0.0	0.0	**44.0**
**Providing information about how your actions are improving the economy**	55.6	18.8	5.4	0.0	**38.3**
**Providing information about how your actions will help things 'get back to normal' quicker**	65.6	14.5	1.7	0.0	**45.4**

Supplementary Table 7a. To what extent do you believe that the measures asked of you by your government or health authority are important to prevent and/or reduce the spread? (Adherence profile definitions in Figure 2.)

	Very important	Somewhat important	Not very important	Not important at all	Total
**Adherent in all three (n=185)**	164 (90.6)	16 (8.8)	1 (0.6)	0	181 (100)
**Non-adherence in at least one of three (n=138)**	68 (50.8)	50 (37.3)	9 (6.7)	7 (5.2)	134 (100)
**Non-adherence in all three (n=60)**	13 (22.0)	32 (54.2)	7 (11.9)	7 (11.9)	59 (100)
**Non-adherence of "never" in all three (n=8)**	1 (14.3)	1 (14.3)	2 (28.6)	3 (42.9)	7 (100)
**Total round 2 (n=1056)**	835 (80.9)	169 (16.4)	18 (1.7)	10 (1.0)	1032 (100)

Supplementary Table 7b. What do you think of the actions taken by your government or health authority to prevent and/or reduce the spread of COVID-19?

	Too strict	About right	Too lenient	Total
**Adherent in all three (n=185)**	3 (1.6)	146 (79.8)	34 (18.6)	183 (100)
**Non-adherence in at least one of three (n=138)**	18 (13.2)	90 (66.2)	28 (20.6)	136 (100)
**Non-adherence in all three (n=60)**	13 (21.7)	40 (66.7)	7 (11.7)	60 (100)
**Non-adherence of "never" in all three (n=8)**	3 (37.5)	3 (37.5)	2 (25.0)	8 (100)
**Total round 2 (n=1056)**	51 (5.0)	772 (75.0)	206 (20.0)	1029 (100)

Supplementary Table 8. What would be your main concerns around having a COVID-19 test if you had symptoms or were exposed to someone with COVID-19? (Adherence profile definitions in Figure 3.)

	**Adherent**	**Non-adherent**			**Total**
	**Adherent in all three (n=185)**	**Non-adherence in at least one of the three (n=138)**	**Non-adherence in all three (n=60)**	**Non-adherence of "never" in all three (n=8)**	**Round 2 (n=1051)**
	**%**	**%**	**%**	**%**	**%**
**Cost of a test**	11.1	7.3	5.0	12.5	**6.8**
**Inconvenience in obtaining a test**	12.2	19.0	21.7	12.5	**14.8**
**Don't know where to get a test**	11.6	16.8	18.3	12.5	**15.3**
**Having to isolate until results return**	23.2	24.1	25.0	12.5	**19.8**
**Having to request your contacts to isolate**	16.6	11.7	15.0	12.5	**20.7**
**Missing employment until results return**	6.6	10.2	5.0	12.5	**7.4**
**Not having paid sick leave if isolation is needed**	6.6	3.7	5.0	12.5	**5.8**
**Social isolation if physical distancing is recommended**	12.5	7.3	5.0	12.5	**9.6**
